# A Mendelian randomization study of the effect of serum 25-hydroxyvitamin D levels on autoimmune thyroid disease

**DOI:** 10.3389/fimmu.2023.1298708

**Published:** 2024-01-08

**Authors:** Yunfeng Yu, Xinyu Yang, Jingyi Wu, Xueli Shangguan, Siyang Bai, Rong Yu

**Affiliations:** ^1^ The First Hospital of Hunan University of Chinese Medicine, Changsha, Hunan, China; ^2^ College of Chinese Medicine, Hunan University of Chinese Medicine, Changsha, Hunan, China; ^3^ The Third School of Clinical Medicine, Zhejiang Chinese Medical University, Hangzhou, Zhejiang, China

**Keywords:** vitamin D, autoimmune thyroid disease, autoimmune thyroiditis, Graves disease, Mendelian randomization

## Abstract

**Objective:**

The influence of vitamin D on autoimmune thyroid disease (AITD) remains a subject of ongoing debate. This study employs Mendelian randomization (MR) to investigate the causal correlations of serum 25-hydroxyvitamin D (25[OH]D) levels with autoimmune thyroiditis (AIT), autoimmune hyperthyroidism (AIH), and Graves disease (GD).

**Methods:**

Data on single nucleotide polymorphisms related to serum 25(OH)D levels, AIT, AIH, and GD were sourced from UK Biobank and FinnGen. Inverse variance weighted, MR-Egger, and weighted median were employed to test the exposure-outcome causal relationship. Assessments of horizontal pleiotropy, heterogeneity, and stability were performed using the MR-Egger intercept, Cochran’s Q test, and leave-one-out sensitivity analysis, respectively.

**Results:**

The results of MR analysis showed increased serum 25(OH)D levels was associated with a reduced risk of AIT (OR 0.499, 95% CI 0.289 to 0.860, *p* = 0.012) but not causal associated with AIH (OR 0.935, 95% CI 0.695 to 1.256, *p* = 0.654) and GD (OR 0.813, 95% CI 0.635 to 1.040, *p* = 0.100). Intercept analysis showed no horizontal pleiotropy (*p* > 0.05), and Cochran’s Q test showed no heterogeneity (*p* > 0.05). Sensitivity analysis suggested that these results were robust.

**Conclusion:**

An increased serum 25(OH)D level is associated with AIT risk reduction but unrelated to AIH and GD. This finding suggests that vitamin D supplementation can be valuable for preventing and treating AIT.

## 1 Introduction

Autoimmune thyroid disease (AITD), an autoimmune disorder characterized by the breakdown of tolerance in the immune system towards thyroid antigens (1), is one of the most common autoimmune diseases. In recent years, the incidence of AITD has been increasing (2). Reports indicate that AITD affects about 5% of the general population, with a higher prevalence among females (2). The pathogenesis of AITD remains incompletely understood but is generally attributed to immune system dysregulation mediated by genetic and environmental triggering factors, with T cells and B cells infiltration into the thyroid glands being its typical manifestation (3). Hashimoto’s thyroiditis (HT) and Graves disease (GD) are the two most frequently discussed AITD types. HT is the most common autoimmune thyroiditis (AIT), which causes damage to thyroid follicular cells, leading to hypothyroidism (4). GD is the most prevalent form of hyperthyroidism, causing thyroid cell proliferation and excessive thyroid hormone synthesis, resulting in hyperthyroidism (5). Currently, levothyroxine is the primary treatment for HT (6), while antithyroid drugs, radioactive iodine therapy, and surgical treatment are the main strategies for GD (7). Nutrients such as vitamin D and selenium have also been considered to positively impact AITD (8).

Vitamin D is a type of steroid hormone produced from the skin or absorbed from the diet, and its primary function is to regulate calcium phosphate metabolism and promote bone homeostasis (9). A relevant study has shown that vitamin D is related to immune regulation functions, which affects monocyte-mediated innate immune responses and regulates adaptive immune responses by inhibiting antigen-presenting cell functions (10). Vitamin D deficiency is believed to be associated with an increased risk of autoimmune diseases such as rheumatoid arthritis, systemic lupus erythematosus, multiple sclerosis, and others (11). However, its role in AITD is not yet clear. It has been reported that serum 25-hydroxyvitamin D (25[OH]D) levels are closely correlated with autoimmune antibody titers such as thyroid peroxidase antibodies (TPOAb), thyroglobulin antibodies (TGAb), and thyroid stimulating hormone receptor antibodies (TRAb) (12, 13). Vitamin D supplementation therapy may potentially enhance AITD prognosis (14). However, some studies have refuted the benefits of vitamin D supplementation for AITD patients (15, 16). The influence of vitamin D on AITD remains controversial, and the causal relationship between the two needs to be further elucidated.

Mendelian randomization (MR) is an epidemiological method used to study causal relationships between exposure factors and outcome variables (17). MR effectively avoids the influence of confounding factors due to the random nature of the genes (18). This study aims to investigate the causal correlations of serum 25(OH)D levels with AIT, autoimmune hyperthyroidism (AIH), and GD from genetic prediction by MR.

## 2 Materials and methods

### 2.1 Study design

**Figure 1:**
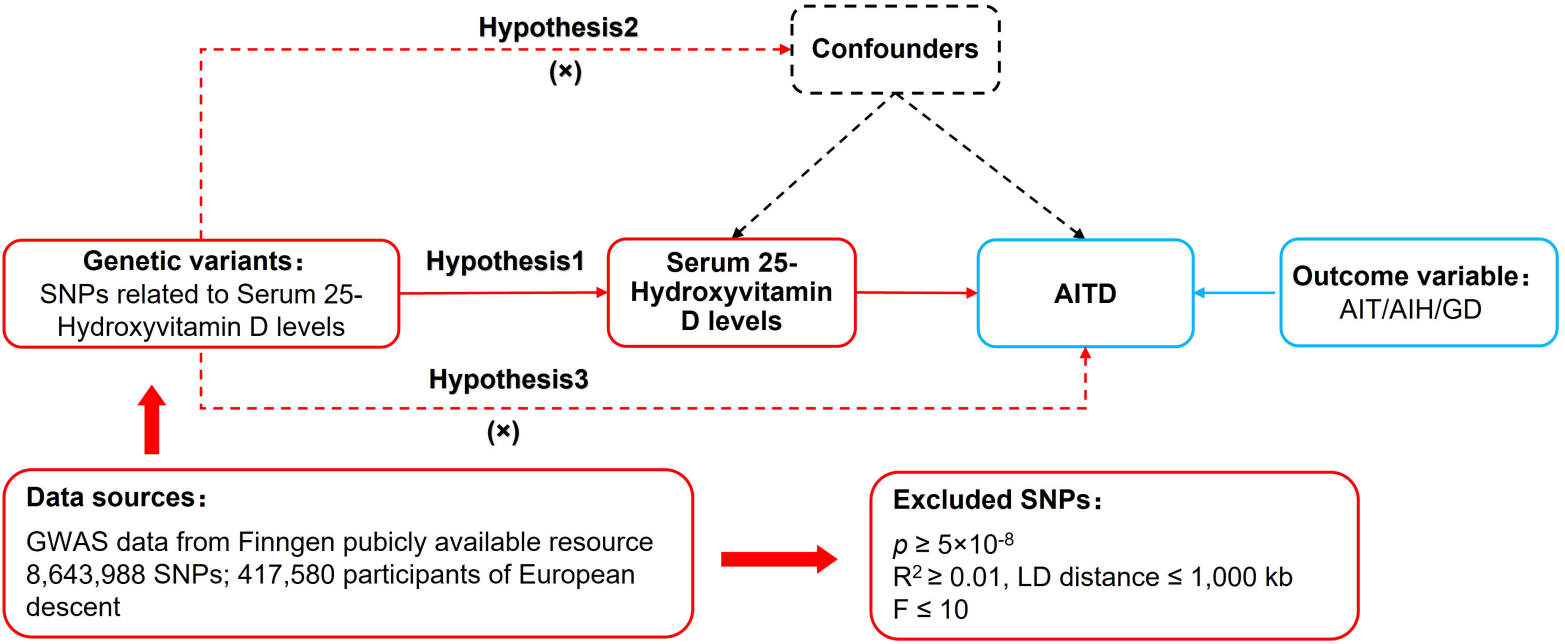
MR design for causal analysis of serum 25(OH)D levels and AITD.

MR relied on three basic assumptions: (1) The relevance assumption: Single nucleotide polymorphisms (SNPs) were closely associated with the exposure factor. (2) The independence assumption: SNPs were independent of confounding factors. (3) The exclusion restriction assumption: SNPs could not affect the outcome variable through pathways other than the exposure factor. The study used a two-sample MR design. “Two-sample” refers to the fact that the exposure factor and the outcome variable came from two different datasets. In this MR analysis, the dataset for serum 25(OH)D levels as an exposure factor was obtained from UK-Biobank, while the datasets for AIT, AIH, and GD as outcome variables were obtained from FinnGen. This approach helped avoid data overfitting problems, horizontal pleiotropy and weak instrumental bias. The MR design process is shown in **Figure 1**.

### 2.2 Data sources

Data on serum 25(OH)D levels, AIT, AIH, and GD were sourced from UK Biobank (www.nealelab.is/uk-biobank) and FinnGen (www.finngen.fi/en). The criteria for defining the status of serum 25(OH)D levels are as follows: Desirable ≥ 75 nmol/L, Sufficient 50 to 74.9 nmol/L, Insufficient 25 to 49.9 nmol/L, Deficient< 25 nmol/L. All data were sourced from publicly available databases, eliminating the need for additional ethical approval.

### 2.3 Selection of genetic instrumental variables

First, SNPs closely associated with exposure factors were screened in the genome-wide association studies (GWAS) database according to a threshold of *p<* 5×10^-8^ to fulfill assumption 1. Second, independent SNPs were screened according to R2< 0.001 and kb = 10,000 to mitigate potential linkage disequilibrium bias. Third, the F-statistics of each SNP were calculated, and the SNPs were further screened according to the parameter of F > 10. The calculation of the F-statistic is as follows: F= [ $R^{2}$ /( $1-R^{2}$ )]*[ (N−K−1 )/ k ]. $R^{2}=2*\left(1-MAF\right)*MAF*\beta^{2}$ . R^2^: The cumulative explained variance of the selected instrumental variables on exposure; MAF: The effect of minor allele frequency; β: The estimated effect of SNP; N: The sample size of the GWAS. Forth, SNPs potentially related to AITD were removed based on PhenoScanner (www.phenoscanner.medschl.cam.ac.uk) and relevant literature to satisfy assumption 2. Finally, duplicates and mismatched SNPs were excluded based on EAF values while harmonizing the allelic orientation of exposure-SNPs and outcome-SNPs. The remaining SNPs were used to perform MR analysis.

### 2.4 Data analysis

The study followed the STROBE-MR guidelines (19). The two-sample MR analysis used the “TwoSampleMR (0.5.7)” package in R 4.3.1 (Lucent Technologies, MurrayHill, State of New Jersey, USA). The primary methods for assessing causal relationships were inverse variance weighting (IVW), MR-Egger, and weighted median. IVW, the main analytical method (20), provides unbiased causal estimates without horizontal pleiotropy and is considered the most informative. MR-Egger and the weighted median are used as complementary methods to MR analysis. MR-Egger can yield effective causal estimates in the presence of some horizontal pleiotropy, while weighted median has a lower sensitivity to outliers and measurement errors.

MR-Pleiotropy Residual Sum and Outlier (MR-PRESSO) was used to remove outlier (*p <* 1) SNPs. The remaining SNPs were used to re-perform the MR analysis and obtain the final results. Horizontal pleiotropy was assessed using MR-Egger’s intercept analysis, with *p* ≥ 0.05 indicating the absence of horizontal pleiotropy, satisfying assumption 3. Heterogeneity was assessed using Cochran’s Q, with *p* ≥ 0.05 indicating the absence of heterogeneity. Sensitivity analysis was performed using the leave-one-out method to assess the robustness of the MR results and identify individual SNPs that significantly affect the combined results.

## 3 Results

### 3.1 GWAS data for serum 25(OH)D levels

Data on serum 25(OH)D levels was obtained from UK-Biobank, which included GWAS data from 417,580 individuals of European descent. UK Biobank provided 113 SNPs closely associated with serum 25(OH)D levels. Among these, 8 SNPs were excluded due to their association with known confounding factors, leaving 105 SNPs included in this study, as shown in **Supplementary Table S1**. Duplicates and mismatched SNPs were excluded based on EAF values while harmonizing the allelic orientation of exposure-SNPs and outcome-SNPs, and the final included SNPs are shown in **Supplementary Tables S2**-**S4**.

### 3.2 GWAS data from AITD

Comprehensive details of the GWAS datasets utilized in this study are presented in **Table 1**. Data for AIT was obtained from the FinnGen database, including 321,192 individuals of European descent (dataset ID: finngen_R9_E4_THYROIDITAUTOIM). Data for AIH was also sourced from FinnGen, including 281,683 European individuals (dataset ID: finngen_R9_AUTOIMMUNE_HYPERTHYROIDISM). Data for GD was obtained from FinnGen, including 377,277 individuals of European descent (dataset ID: finngen_R9_E4_GRAVES_STRICT).

**Table 1:** Details of the GWAS studies included in the Mendelian randomization.

Year	Trait	Population	Sample size	Web source
2020	Serum 25-Hydroxyvitamin D levels	European	417,580	www.nealelab.is/uk-biobank
2023	Autoimmune thyroiditis	European	321,192	www.finngen.fi/en
2023	Autoimmune hyperthyroidism	European	281,683	www.finngen.fi/en
2023	Graves disease	European	377,277	www.finngen.fi/en

### 3.3 Two-Sample MR Analysis Results

**Figure 2:**
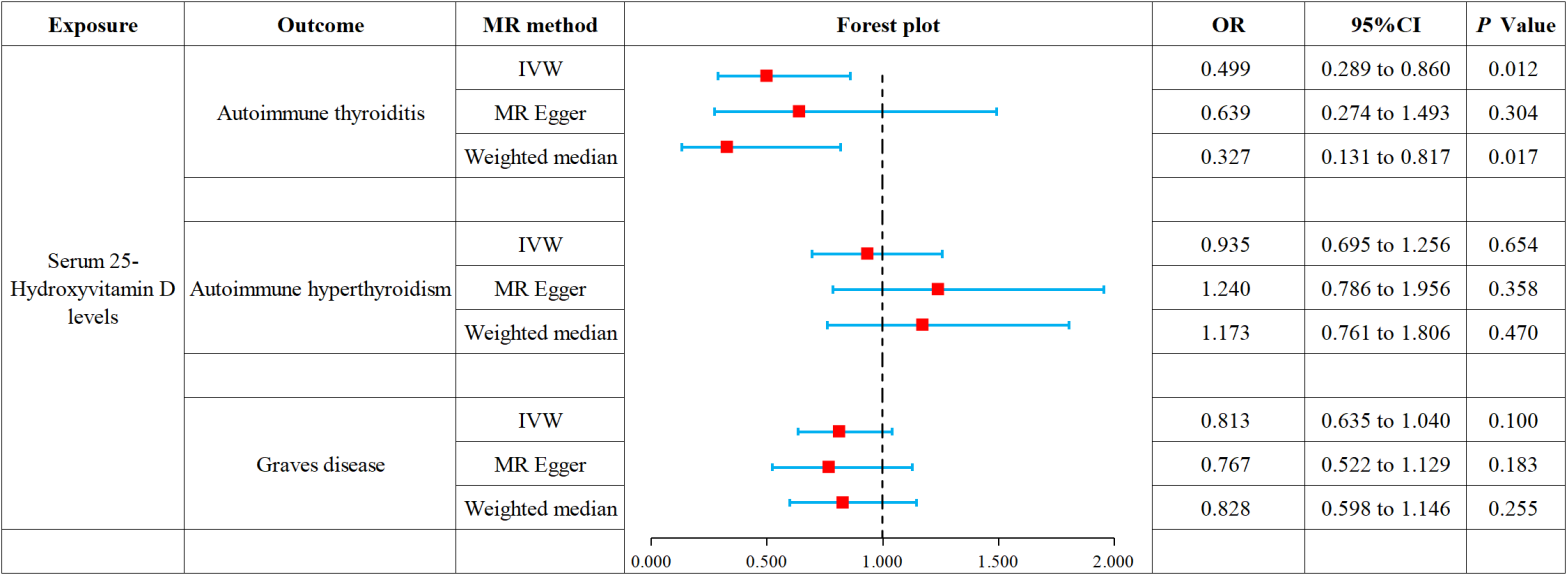
Forest plot of MR analysis on the causal relationship between serum 25(OH)D levels and AITD.

**Figure 3:**
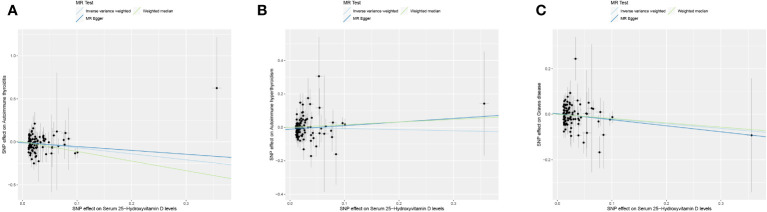
Scatter plot of MR analysis on the causal relationship between serum 25(OH)D levels and AITD. **(A)** AIT, **(B)** AIH, **(C)** GD. *AITD, autoimmune thyroid disease; AIT, autoimmune thyroiditis; AIH, autoimmune hyperthyroidism; GD, Graves disease.

**Figure 4:**
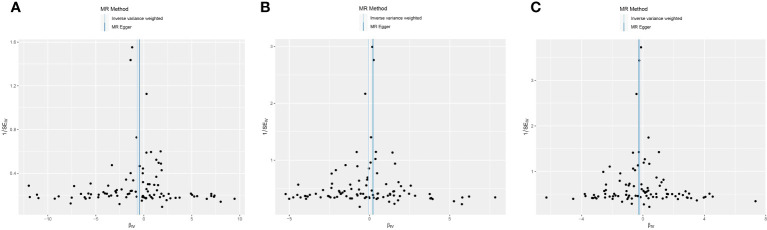
Funnel plot of heterogeneity analysis on serum 25(OH)D levels and AITD. **(A)** AIT, **(B)** AIH, **(C)** GD. *AITD, autoimmune thyroid disease; AIT, autoimmune thyroiditis; AIH, autoimmune hyperthyroidism; GD, Graves disease.

**Figure 5:**
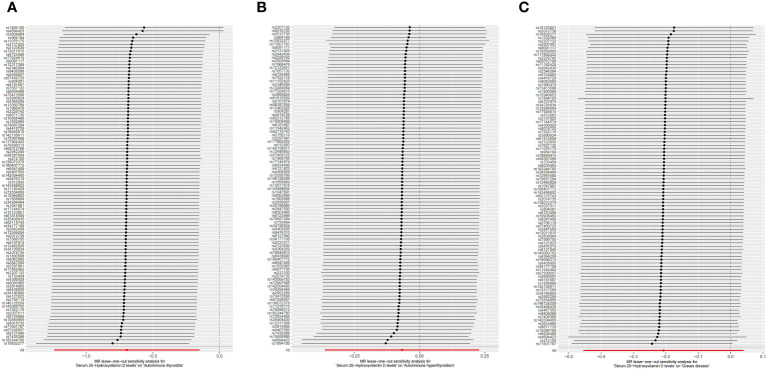
Leave-one-out sensitivity analysis on serum 25(OH)D levels and AITD. **(A)** AIT, **(B)** AIH, **(C)** GD. *AITD, autoimmune thyroid disease; AIT, autoimmune thyroiditis; AIH, autoimmune hyperthyroidism; GD, Graves disease.

This study conducted MR to analyze the causal correlations of serum 25(OH)D levels with AIT, AIH, and GD. The forest plot of the MR analysis is shown in **Figure 2**, and the effect estimates for each SNP are displayed in **Figure 3**. MR-Egger intercept analysis is presented in **Supplementary Table S5**, heterogeneity test results are in **Figure 4**; **Supplementary Table S6**, and sensitivity analysis is in **Figure 5**.

#### 3.3.1 AIT

IVW (OR 0.499, 95% CI 0.289 to 0.860, *p* = 0.012) and weighted median (OR 0.327, 95% CI 0.131 to 0.817, *p* = 0.017) revealed that increased serum 25(OH)D levels are associated with a reduced risk of AIT. At the same time, MR-Egger (OR 0.639, 95% CI 0.274 to 1.493, *p* = 0.304) did not observe such a causal relationship. Intercept analysis indicated the absence of horizontal pleiotropy (*p* = 0.455), heterogeneity test showed no significant heterogeneity (*p* = 0.734), and sensitivity analysis suggested the results were robust.

#### 3.3.2 AIH

All three analytical methods showed no significant causal relationship between serum 25(OH)D levels and AIH: IVW (OR 0.935, 95% CI 0.695 to 1.256, *p* = 0.654), MR-Egger (OR 1.240, 95% CI 0.786 to 1.956, *p* 0.358) and weighted median (OR 1.173, 95% CI 0.761 to 1.806, *p* = 0.470). Intercept analysis indicated the absence of horizontal pleiotropy (*p* = 0.116), heterogeneity test showed no significant heterogeneity (*p*=0.264), and sensitivity analysis suggested the results were robust.

#### 3.3.3 GD

All three analytical methods showed no significant causal relationship between serum 25(OH)D levels and GD: IVW (OR 0.813, 95% CI 0.635 to 1.040, *p* = 0.100), MR-Egger (OR 0.767, 95% CI 0.522 to 1.129, *p* = 0.183) and weighted median (OR 0.828, 95%CI 0.598 to 1.146, *p* = 0.255). Intercept analysis indicated the absence of horizontal pleiotropy (*p* = 0.706), heterogeneity test showed no significant heterogeneity (*p* = 0.112), and sensitivity analysis suggested the results were robust.

## 4 Discussion

AITD is a significant risk factor for thyroid cancer (21). Recent studies have shown that vitamin D deficiency increases the risk and difficulty in treating AITD (22), which may be related to its role in modulating the adaptive immune response (23). However, other reports suggest that vitamin D levels are not associated with AITD (15, 16), and supraphysiological levels of vitamin D supplementation may even increase the risk of death (24). To gain a clearer understanding of the role of vitamin D on AITD, this study used MR to analyze the causal relationship of serum 25(OH)D levels with AIT, AIH, and GD.

Our study results indicated increased serum 25(OH)D levels was associated with a reduced risk of AIT but not causal associated with AIH and GD. These results were free of horizontal pleiotropy and heterogeneity, and sensitivity analysis suggested they were robust. Since all our data were derived from Europeans, this study primarily illustrated the link between elevated serum 25(OH)D levels and reduced AIT risk in Europeans. Additionally, we found that increased serum 25(OH)D levels were associated with a lower risk of AIT but not GD, probably due to the different pathogenesis and specific antibodies of the two.

HT is one of the main types of ATID and the most common form of AIT (25). Our study results indicated an association between elevated serum 25(OH)D levels and a diminished AIT risk. Unal AD et al. (26) conducted a cross-sectional investigation, revealing markedly lower serum 25(OH)D levels in HT patients compared to healthy individuals. At the same time, they found serum 25(OH)D levels exhibited a negative correlation with TPOAb and TGAb titers in HT patients. A subsequent study confirmed this conclusion and reported a substantial association between vitamin D deficiency and notable thyroid dysfunction among individuals with HT (27). A study of Polish women showed a strong negative correlation between thyroid stimulating hormone (TSH) and vitamin D levels across healthy individuals, HT patients, and hypothyroid patients, while TPOAb and TGAb exhibited a weak negative correlation with vitamin D levels (12). Bozkurt NC et al. (28) highlighted that the severity of vitamin D deficiency was not only related to antibody levels but also to the HT duration and thyroid volume. Therefore, vitamin D deficiency may be a potential risk factor for AIT, and vitamin D supplementation may benefit patients with AIT.

A clinical study by Ucan B et al. (29) found that vitamin D supplementation therapy at 50,000 IU per week for eight weeks can slow down thyroid dysfunction in HT patients and reduce their cardiovascular risk. In a double-blind randomized controlled trial, Chahardoli R et al. (30) reported that weekly supplementation with 50,000 IU of vitamin D reduced TSH and TGAb levels in female HT patients, attenuating their disease activity. Meta-analysis suggested that vitamin D supplementation ≤3 months effectively reduced TGAb titers in HT patients but had no benefit in TPOAb titers, while supplementation ≥3 months reduced both TGAb and TPOAb titers, indicating that long-term vitamin D intake may have more significant benefits to HT patients (31).

However, some studies believe that HT is unrelated to higher vitamin D deficiency rates (32). Pani MA et al. (33), through a study on polymorphic vitamin D binding protein (DBP), found that DBP allelic variation did not confer susceptibility to HT in people of Caucasian ancestry. Cvek M et al. (16), through observation of CROHT biobank, found that vitamin D levels showed no correlation with the presence of HT. Anaraki PV et al. (34) reported that vitamin D supplementation therapy (50,000 IU per week for 12 weeks) did not improve metabolism-related parameters such as glucose, lipids, albumin, and electrolytes in HT patients with vitamin D deficiency. Although the causal relationship between vitamin D and AIT remains controversial, most studies support the connection between vitamin D deficiency and an elevated AIT risk. These findings align with our study results, suggesting that vitamin D supplementation may mitigate the risk and improve the prognosis of AIT.

The effect of vitamin D on AIT may be related to the inhibition of T and B lymphocytes. Vitamin D inhibits dendritic cell-dependent T-cell activation and reduces HLA II gene expression to suppress pro-inflammatory factor expression (10). The vitamin also increases the number of Tregs while inhibiting the differentiation of naive T cells into Th17 (35).

GD is one of the main AITD types and is the most common form of hyperthyroidism (36). Our study found that serum 25(OH)D levels are not associated with AIH and GD risk, similar to previous reports. A clinical study in China showed that serum 25(OH)D levels were relatively deficient in HT patients but similar to healthy individuals in GD patients (37). Although cross-sectional studies in India by Mangaraj S et al. (38) and Planck T et al. (39) reported lower serum 25(OH)D levels in GD patients, they did not support a correlation between these levels and thyroid hormones or TRAb. Research by Yasuda T et al. (40) further confirmed that serum 25(OH)D levels affected only thyroid volume but were not associated with thyroid function or TRAb. Conversely, Zhang H et al. (13) found that although vitamin D levels were unrelated to FT3, FT4, TSH, TGAb, TPOAb, and other indicators in GD patients, they were closely related to TRAb titers. They suggested that vitamin D deficiency might be linked to heightened autoimmune responses in GD patients (13). Veneti S et al. (41) pointed out from a genetic perspective that the vitamin D receptor gene polymorphism was associated with GD, and the TT subtype of TaqI polymorphism was linked to heightened GD susceptibility. Xu MY et al.’s (42) meta-analysis suggested that low vitamin D status may increase the risk of developing GD. These pieces of evidence point to a possible association of serum 25(OH)D levels with GD risk.

However, previous studies have suggested that vitamin D supplementation is ineffective in treating GD. The DAGMAR Trial (15) showed that supplementing vitamin D did not significantly benefit GD patients with normal or insufficient vitamin D levels. Cho YY et al.’s (43) report showed that vitamin D supplementation did not significantly affect the recurrence rate of GD within one year of discontinuing antithyroid drugs. Clinical trials by Grove-Laugesen D et al. (44, 45) found that vitamin D supplementation of 2,800 IU per day for nine months did not achieve a positive effect on pulse wave velocity and even had adverse effects on the recovery of muscle performance. In summary, the association between vitamin D and GD remains controversial and needs to be explored in more studies in the future. There is insufficient evidence to suggest that vitamin D supplementation benefits GD, so we do not recommend it for GD patients without vitamin D deficiency.

Interestingly, we found that increased serum vitamin D levels were associated with a reduced risk of AIT but not with a GD risk. This difference may be related to the different pathogenesis of the two. Studies have shown that AIT is dominated by an autoimmune response mediated by Th1 cells (46), whereas GD is dominated by a humoral response mediated by Th2 cells (47). Vitamin D can inhibit the proliferation and differentiation of Th1 cells, and it can reduce the risk of AIT by suppressing the Th1-dominated immune response (48).

Of the 105 SNPs for serum 25(OH)D levels included in this study, only ten SNPs have been reported in the literature, as shown in **Supplementary Table S7**. Of these, rs801872, located in the SEC23A gene, was reported to be significantly associated with 25(OH)D levels (49), and the remaining nine SNPs were not reported to be associated with vitamin D and its receptor. Moreover, the published literature did not report the relevance of these SNPs to AITD. More studies are needed in the future to explore the role of these SNPs in the pathogenesis of AIT and to search for the key loci of vitamin D regulation of AIT.

Our study also has certain limitations. First, because the GWAS database only contains datasets for AIT, AIH, and GD, this study can only explain how serum 25(OH)D levels relate to them and does not apply to all types of AITD. Second, because the database lacks matched data on Asian ancestry and African ancestry, the entirety of our data was derived from Europeans, which leads to the possibility that the results of this study may not apply to other races. Third, this study was conducted based on the exposure factor serum 25(OH)D levels for the outcome variables AIT, AIH, and GD. Therefore, it can only describe the effect of serum 25(OH)D levels on disease risk and does not apply to antibody levels.

Given these limitations, we look forward to future research improvements: First, conducting some stratified experiments. In these experiments, relevant variables should be controlled to investigate the effects of different doses and durations of vitamin D supplementation on thyroid hormone levels and autoimmune antibody levels in different AITD patients. Second, establishing research centers in multiple continents and countries to investigate the effects of vitamin D levels on AITD patients of different races to provide a more comprehensive data source for MR studies.

## 5 Conclusion

This MR analysis suggests that elevated serum 25(OH)D levels correlate with a decreased AIT risk but are not associated with AIH and GD. Vitamin D supplementation may help mitigate the risk and enhance the prognosis of AIT. In forthcoming studies, further investigation is warranted to delve into the causal relationship and underlying mechanisms linking vitamin D to AITD.

## Data availability statement

The original contributions presented in the study are included in the article/**Supplementary Material**. Further inquiries can be directed to the corresponding author.

## Ethics statement

Ethical approval was not required for the study involving humans in accordance with the local legislation and institutional requirements. Written informed consent to participate in this study was not required from the participants or the participants’ legal guardians/next of kin in accordance with the national legislation and the institutional requirements.

## Author contributions

YY: Conceptualization, Data curation, Supervision, Writing – original draft. XY: Methodology, Supervision, Writing – original draft. JW: Data curation, Methodology, Writing – original draft. XS: Data curation, Formal analysis, Writing – original draft. SB: Formal analysis, Writing – original draft. RY: Formal analysis, Writing – review & editing.
