# Supplementary material for: A Mendelian randomization study of the effect of serum 25-hydroxyvitamin D levels on autoimmune thyroid disease
Source: Front Immunol. 2024 Jan 8;14:1298708. doi: 10.3389/fimmu.2023.1298708 (PMC10800945; doi:10.3389/fimmu.2023.1298708)
Supplement: Supplementary file 1 [file Table_1.docx]

**Supplementary Table S1** Characteristics of instrumental variables for serum 25-hydroxyvitamin D levels

|  | **SNP** | **EA** | **OA** | **Samplesize** | **SE** | **β** | **id.exposure** | **EAF** | ***P* value** | **R^2^** | **F - statistic** |
| --- | --- | --- | --- | --- | --- | --- | --- | --- | --- | --- | --- |
| 1 | rs6671730 | A | G | 417580 | 0.00197958 | -0.015115 | ebi-a-GCST90000615 | 0.434289 | 2.25E-14 | 0.000112259 | 46.88199859 |
| 2 | rs35408430 | T | C | 417580 | 0.00206739 | -0.0213322 | ebi-a-GCST90000615 | 0.342171 | 5.82E-25 | 0.00020486 | 85.56262344 |
| 3 | rs79598313 | T | C | 417580 | 0.00648419 | -0.0370228 | ebi-a-GCST90000615 | 0.023367 | 1.13E-08 | 6.26E-05 | 26.12568291 |
| 4 | rs7522116 | T | C | 417580 | 0.00199431 | -0.0126086 | ebi-a-GCST90000615 | 0.566284 | 2.58E-10 | 7.81E-05 | 32.61181657 |
| 5 | rs512083 | C | T | 417580 | 0.00197554 | 0.0122018 | ebi-a-GCST90000615 | 0.460817 | 6.56E-10 | 7.40E-05 | 30.8967092 |
| 6 | rs2131925 | T | G | 417580 | 0.00205242 | -0.0222318 | ebi-a-GCST90000615 | 0.643589 | 2.43E-27 | 0.000226746 | 94.70546848 |
| 7 | rs12123821 | T | C | 417580 | 0.00460381 | 0.0772297 | ebi-a-GCST90000615 | 0.047541 | 3.71E-63 | 0.000540149 | 225.676091 |
| 8 | rs61815559 | T | A | 417580 | 0.00564426 | 0.0845633 | ebi-a-GCST90000615 | 0.031417 | 9.60E-51 | 0.000435207 | 181.8117947 |
| 9 | rs10908469 | C | A | 417580 | 0.00221124 | 0.0158902 | ebi-a-GCST90000615 | 0.269726 | 6.67E-13 | 9.95E-05 | 41.54111842 |
| 10 | rs79687284 | C | G | 417580 | 0.00537042 | -0.0295438 | ebi-a-GCST90000615 | 0.034551 | 3.77E-08 | 5.82E-05 | 24.31731181 |
| 11 | rs2642439 | G | A | 417580 | 0.00211201 | -0.0145212 | ebi-a-GCST90000615 | 0.68503 | 6.18E-12 | 9.10E-05 | 38.0006409 |
| 12 | rs11122455 | G | C | 417580 | 0.00201492 | 0.0126509 | ebi-a-GCST90000615 | 0.614056 | 3.42E-10 | 7.59E-05 | 31.67930684 |
| 13 | rs6547409 | T | C | 417580 | 0.00452445 | 0.0279437 | ebi-a-GCST90000615 | 0.049758 | 6.57E-10 | 7.38E-05 | 30.83646836 |
| 14 | rs13011615 | T | A | 417580 | 0.00288288 | 0.0169587 | ebi-a-GCST90000615 | 0.135062 | 4.04E-09 | 6.72E-05 | 28.06080062 |
| 15 | rs58387006 | C | A | 417580 | 0.0023619 | -0.0137174 | ebi-a-GCST90000615 | 0.221848 | 6.33E-09 | 6.50E-05 | 27.13059903 |
| 16 | rs6723486 | T | C | 417580 | 0.00202854 | -0.0111173 | ebi-a-GCST90000615 | 0.610563 | 4.24E-08 | 5.88E-05 | 24.54479879 |
| 17 | rs6724965 | G | A | 417580 | 0.00259881 | -0.0165826 | ebi-a-GCST90000615 | 0.171461 | 1.76E-10 | 7.81E-05 | 32.62759749 |
| 18 | rs7569755 | A | G | 417580 | 0.00217782 | 0.0132378 | ebi-a-GCST90000615 | 0.290628 | 1.21E-09 | 7.23E-05 | 30.1746411 |
| 19 | rs1047891 | A | C | 417580 | 0.00210766 | -0.0125534 | ebi-a-GCST90000615 | 0.315755 | 2.58E-09 | 6.81E-05 | 28.4368694 |
| 20 | rs2012736 | A | C | 417580 | 0.00360843 | -0.0481921 | ebi-a-GCST90000615 | 0.080848 | 1.10E-40 | 0.000345174 | 144.186918 |
| 21 | rs113642272 | T | G | 417580 | 0.00206013 | -0.019613 | ebi-a-GCST90000615 | 0.635926 | 1.73E-21 | 0.000178121 | 74.39252042 |
| 22 | rs6438900 | G | C | 417580 | 0.00225847 | 0.0133459 | ebi-a-GCST90000615 | 0.257991 | 3.44E-09 | 6.82E-05 | 28.47781629 |
| 23 | rs34186890 | G | A | 417580 | 0.00224148 | -0.0149926 | ebi-a-GCST90000615 | 0.25971 | 2.25E-11 | 8.64E-05 | 36.0952129 |
| 24 | rs78649910 | A | T | 417580 | 0.00320205 | -0.0193309 | ebi-a-GCST90000615 | 0.106174 | 1.57E-09 | 7.09E-05 | 29.61922703 |
| 25 | rs4364259 | A | G | 417580 | 0.0024674 | 0.0165067 | ebi-a-GCST90000615 | 0.202159 | 2.23E-11 | 8.79E-05 | 36.7058873 |
| 26 | rs7439366 | C | T | 417580 | 0.00196647 | -0.0315734 | ebi-a-GCST90000615 | 0.455513 | 5.20E-58 | 0.000494494 | 206.5919567 |
| 27 | rs4306928 | C | T | 417580 | 0.00263067 | 0.0250598 | ebi-a-GCST90000615 | 0.832347 | 1.63E-21 | 0.000175267 | 73.2006064 |
| 28 | rs4694423 | A | C | 417580 | 0.00199111 | -0.100874 | ebi-a-GCST90000615 | 0.416466 | 0 | 0.004945773 | 2075.511093 |
| 29 | rs71601787 | A | G | 417580 | 0.00210912 | 0.0419281 | ebi-a-GCST90000615 | 0.326737 | 6.13E-88 | 0.000773434 | 323.2191352 |
| 30 | rs7657132 | G | A | 417580 | 0.00213658 | -0.0140781 | ebi-a-GCST90000615 | 0.316685 | 4.43E-11 | 8.58E-05 | 35.82130458 |
| 31 | rs6834488 | T | C | 417580 | 0.00199028 | -0.0146317 | ebi-a-GCST90000615 | 0.423712 | 1.96E-13 | 0.000104551 | 43.6629355 |
| 32 | rs189407772 | G | A | 417580 | 0.00661678 | 0.0530974 | ebi-a-GCST90000615 | 0.022692 | 1.02E-15 | 0.000125049 | 52.22430356 |
| 33 | rs3890624 | G | A | 417580 | 0.00203846 | 0.0112736 | ebi-a-GCST90000615 | 0.395101 | 3.19E-08 | 6.07E-05 | 25.36940188 |
| 34 | rs1966478 | C | T | 417580 | 0.00213261 | -0.0123541 | ebi-a-GCST90000615 | 0.692961 | 6.92E-09 | 6.49E-05 | 27.12191641 |
| 35 | rs72834856 | G | T | 417580 | 0.00379173 | -0.0235978 | ebi-a-GCST90000615 | 0.072057 | 4.86E-10 | 7.45E-05 | 31.09857267 |
| 36 | rs9467550 | G | A | 417580 | 0.00319549 | -0.0188955 | ebi-a-GCST90000615 | 0.105238 | 3.36E-09 | 6.72E-05 | 28.07978013 |
| 37 | rs143069752 | A | T | 417580 | 0.00392801 | 0.0224092 | ebi-a-GCST90000615 | 0.067349 | 1.16E-08 | 6.31E-05 | 26.344989 |
| 38 | rs9476310 | T | C | 417580 | 0.00197015 | 0.0117234 | ebi-a-GCST90000615 | 0.511339 | 2.67E-09 | 6.87E-05 | 28.68277719 |
| 39 | rs2608984 | T | A | 417580 | 0.00264074 | -0.0208623 | ebi-a-GCST90000615 | 0.165239 | 2.79E-15 | 0.000120068 | 50.14398386 |
| 40 | rs17144574 | C | T | 417580 | 0.00233815 | -0.01526 | ebi-a-GCST90000615 | 0.233706 | 6.73E-11 | 8.34E-05 | 34.83198182 |
| 41 | rs7784802 | T | A | 417580 | 0.00204009 | 0.0137867 | ebi-a-GCST90000615 | 0.360988 | 1.40E-11 | 8.77E-05 | 36.62082472 |
| 42 | rs1858889 | C | A | 417580 | 0.00196228 | 0.0111372 | ebi-a-GCST90000615 | 0.501533 | 1.38E-08 | 6.20E-05 | 25.89897068 |
| 43 | rs2346264 | C | A | 417580 | 0.00239864 | -0.0147004 | ebi-a-GCST90000615 | 0.782699 | 8.86E-10 | 7.35E-05 | 30.69830097 |
| 44 | rs804281 | G | A | 417580 | 0.00199025 | 0.0157516 | ebi-a-GCST90000615 | 0.583598 | 2.48E-15 | 0.000120589 | 50.36118386 |
| 45 | rs34726834 | T | C | 417580 | 0.00227206 | 0.0149657 | ebi-a-GCST90000615 | 0.252025 | 4.49E-11 | 8.44E-05 | 35.26379561 |
| 46 | rs57459725 | G | C | 417580 | 0.00289594 | -0.0176615 | ebi-a-GCST90000615 | 0.132913 | 1.07E-09 | 7.19E-05 | 30.0250776 |
| 47 | rs12056768 | G | T | 417580 | 0.00199283 | -0.021949 | ebi-a-GCST90000615 | 0.582891 | 3.27E-28 | 0.000234259 | 97.84434773 |
| 48 | rs13284054 | C | T | 417580 | 0.00308554 | 0.0185546 | ebi-a-GCST90000615 | 0.117755 | 1.82E-09 | 7.15E-05 | 29.87242145 |
| 49 | rs9409266 | A | G | 417580 | 0.00283662 | -0.0184021 | ebi-a-GCST90000615 | 0.861121 | 8.74E-11 | 8.10E-05 | 33.82503987 |
| 50 | rs532436 | A | G | 417580 | 0.00252848 | -0.0169916 | ebi-a-GCST90000615 | 0.183796 | 1.82E-11 | 8.66E-05 | 36.17500109 |
| 51 | rs3925446 | A | G | 417580 | 0.00245773 | 0.0162616 | ebi-a-GCST90000615 | 0.199109 | 3.68E-11 | 8.43E-05 | 35.22046514 |
| 52 | rs4418728 | T | G | 417580 | 0.00196937 | 0.0121203 | ebi-a-GCST90000615 | 0.45168 | 7.54E-10 | 7.28E-05 | 30.38721475 |
| 53 | rs2297991 | C | T | 417580 | 0.00218295 | 0.0120361 | ebi-a-GCST90000615 | 0.720529 | 3.51E-08 | 5.83E-05 | 24.36423238 |
| 54 | rs117862422 | C | T | 417580 | 0.00850362 | -0.0541519 | ebi-a-GCST90000615 | 0.013588 | 1.91E-10 | 7.86E-05 | 32.82789454 |
| 55 | rs143488652 | G | A | 417580 | 0.00855038 | -0.0629543 | ebi-a-GCST90000615 | 0.013389 | 1.80E-13 | 0.000104707 | 43.72783493 |
| 56 | rs138072379 | T | C | 417580 | 0.00720764 | 0.0542072 | ebi-a-GCST90000615 | 0.018983 | 5.44E-14 | 0.000109442 | 45.70571101 |
| 57 | rs182244780 | A | G | 417580 | 0.00864543 | -0.356294 | ebi-a-GCST90000615 | 0.013019 | 0 | 0.003262372 | 1366.753481 |
| 58 | rs10832277 | A | G | 417580 | 0.00204399 | -0.075469 | ebi-a-GCST90000615 | 0.368545 | 2.02E-298 | 0.002650941 | 1109.917105 |
| 59 | rs146128209 | G | A | 417580 | 0.00383087 | -0.057293 | ebi-a-GCST90000615 | 0.070861 | 1.43E-50 | 0.000432236 | 180.5703574 |
| 60 | rs57938057 | C | T | 417580 | 0.00745585 | -0.0787942 | ebi-a-GCST90000615 | 0.01768 | 4.19E-26 | 0.000215652 | 90.07100741 |
| 61 | rs1894100 | T | G | 417580 | 0.00253888 | -0.096459 | ebi-a-GCST90000615 | 0.184194 | 0 | 0.002796262 | 1170.931778 |
| 62 | rs72997688 | G | A | 417580 | 0.00422905 | 0.0243759 | ebi-a-GCST90000615 | 0.057047 | 8.22E-09 | 6.39E-05 | 26.69559132 |
| 63 | rs733454 | T | C | 417580 | 0.00328005 | 0.0195549 | ebi-a-GCST90000615 | 0.099568 | 2.50E-09 | 6.86E-05 | 28.63381982 |
| 64 | rs964184 | C | G | 417580 | 0.00289883 | 0.0431884 | ebi-a-GCST90000615 | 0.868375 | 3.37E-50 | 0.000426393 | 178.1282295 |
| 65 | rs2847500 | A | G | 417580 | 0.00298092 | -0.0221038 | ebi-a-GCST90000615 | 0.123505 | 1.22E-13 | 0.000105779 | 44.17549399 |
| 66 | rs12317268 | G | A | 417580 | 0.00274181 | -0.0206326 | ebi-a-GCST90000615 | 0.150996 | 5.26E-14 | 0.000109147 | 45.58249643 |
| 67 | rs2171427 | A | G | 417580 | 0.00273753 | -0.0154101 | ebi-a-GCST90000615 | 0.154903 | 1.81E-08 | 6.22E-05 | 25.96401856 |
| 68 | rs11182428 | C | T | 417580 | 0.00196317 | -0.0123489 | ebi-a-GCST90000615 | 0.520012 | 3.17E-10 | 7.61E-05 | 31.79076369 |
| 69 | rs8181687 | A | G | 417580 | 0.00198761 | 0.0116958 | ebi-a-GCST90000615 | 0.57904 | 4.00E-09 | 6.67E-05 | 27.84875799 |
| 70 | rs10859995 | C | T | 417580 | 0.00198939 | -0.0399464 | ebi-a-GCST90000615 | 0.58262 | 1.11E-89 | 0.000776073 | 324.3225134 |
| 71 | rs73413596 | C | T | 417580 | 0.00376674 | 0.0237483 | ebi-a-GCST90000615 | 0.073872 | 2.89E-10 | 7.72E-05 | 32.22678977 |
| 72 | rs34284484 | G | T | 417580 | 0.002169 | -0.0123129 | ebi-a-GCST90000615 | 0.285596 | 1.37E-08 | 6.19E-05 | 25.83516127 |
| 73 | rs8018720 | C | G | 417580 | 0.0025687 | -0.0383637 | ebi-a-GCST90000615 | 0.823309 | 1.95E-50 | 0.000428202 | 178.8841555 |
| 74 | rs142004400 | C | A | 417580 | 0.00537846 | -0.0332364 | ebi-a-GCST90000615 | 0.034542 | 6.43E-10 | 7.37E-05 | 30.76864877 |
| 75 | rs2756119 | A | G | 417580 | 0.0020411 | 0.0121881 | ebi-a-GCST90000615 | 0.383266 | 2.35E-09 | 7.02E-05 | 29.32704521 |
| 76 | rs1532085 | G | A | 417580 | 0.0020142 | 0.0260168 | ebi-a-GCST90000615 | 0.61488 | 3.62E-38 | 0.000320571 | 133.9063114 |
| 77 | rs1800588 | T | C | 417580 | 0.00238443 | -0.0329963 | ebi-a-GCST90000615 | 0.215208 | 1.50E-43 | 0.000367768 | 153.6281569 |
| 78 | rs62012766 | C | T | 417580 | 0.00269511 | -0.0181582 | ebi-a-GCST90000615 | 0.157114 | 1.61E-11 | 8.73E-05 | 36.46992333 |
| 79 | rs2123930 | A | G | 417580 | 0.00219611 | -0.014114 | ebi-a-GCST90000615 | 0.279361 | 1.30E-10 | 8.02E-05 | 33.49547996 |
| 80 | rs77924615 | A | G | 417580 | 0.00251244 | -0.0144881 | ebi-a-GCST90000615 | 0.193446 | 8.09E-09 | 6.55E-05 | 27.35345101 |
| 81 | rs8063565 | C | G | 417580 | 0.00221722 | 0.0123302 | ebi-a-GCST90000615 | 0.733599 | 2.68E-08 | 5.94E-05 | 24.81579755 |
| 82 | rs11076175 | G | A | 417580 | 0.00256672 | 0.023978 | ebi-a-GCST90000615 | 0.178375 | 9.46E-21 | 0.000168525 | 70.38406476 |
| 83 | rs11542462 | A | G | 417580 | 0.00287287 | -0.0232523 | ebi-a-GCST90000615 | 0.134348 | 5.79E-16 | 0.000125758 | 52.52046984 |
| 84 | rs34177108 | A | C | 417580 | 0.00223709 | -0.0122879 | ebi-a-GCST90000615 | 0.267502 | 3.96E-08 | 5.92E-05 | 24.71053327 |
| 85 | rs12949853 | A | G | 417580 | 0.00253426 | 0.0144344 | ebi-a-GCST90000615 | 0.806726 | 1.23E-08 | 6.50E-05 | 27.13268574 |
| 86 | rs61003750 | C | G | 417580 | 0.0021399 | -0.0120389 | ebi-a-GCST90000615 | 0.306761 | 1.85E-08 | 6.16E-05 | 25.74252385 |
| 87 | rs2952289 | T | C | 417580 | 0.00245386 | 0.0178756 | ebi-a-GCST90000615 | 0.798034 | 3.22E-13 | 0.000103003 | 43.01632022 |
| 88 | rs2659007 | A | G | 417580 | 0.00198953 | 0.0114099 | ebi-a-GCST90000615 | 0.450974 | 9.75E-09 | 6.45E-05 | 26.92177537 |
| 89 | rs8091117 | A | C | 417580 | 0.0039672 | -0.02514 | ebi-a-GCST90000615 | 0.065264 | 2.34E-10 | 7.71E-05 | 32.20284934 |
| 90 | rs4121823 | A | T | 417580 | 0.00273512 | -0.0185043 | ebi-a-GCST90000615 | 0.845326 | 1.33E-11 | 8.95E-05 | 37.3932626 |
| 91 | rs2037511 | A | G | 417580 | 0.00263857 | 0.0170377 | ebi-a-GCST90000615 | 0.165978 | 1.07E-10 | 8.04E-05 | 33.56235894 |
| 92 | rs142158911 | A | G | 417580 | 0.00309689 | 0.0266711 | ebi-a-GCST90000615 | 0.114629 | 7.17E-18 | 0.000144388 | 60.30203508 |
| 93 | rs12462826 | A | G | 417580 | 0.00204832 | -0.0131168 | ebi-a-GCST90000615 | 0.368713 | 1.52E-10 | 8.01E-05 | 33.44825619 |
| 94 | rs8107974 | T | A | 417580 | 0.00369585 | 0.0386164 | ebi-a-GCST90000615 | 0.076248 | 1.49E-25 | 0.000210067 | 87.73770355 |
| 95 | rs3814995 | T | C | 417580 | 0.00211677 | -0.0126428 | ebi-a-GCST90000615 | 0.31156 | 2.33E-09 | 6.86E-05 | 28.63463899 |
| 96 | rs212100 | C | T | 417580 | 0.00264866 | -0.065701 | ebi-a-GCST90000615 | 0.835997 | 7.84E-136 | 0.00118367 | 494.860104 |
| 97 | rs62115743 | T | C | 417580 | 0.00361625 | 0.0274464 | ebi-a-GCST90000615 | 0.081513 | 3.21E-14 | 0.000112798 | 47.10719702 |
| 98 | rs11606 | G | C | 417580 | 0.00201965 | 0.0112201 | ebi-a-GCST90000615 | 0.425178 | 2.77E-08 | 6.15E-05 | 25.69756377 |
| 99 | rs2207132 | A | G | 417580 | 0.00549169 | -0.0386454 | ebi-a-GCST90000615 | 0.03289 | 1.96E-12 | 9.50E-05 | 39.67749363 |
| 100 | rs1841850 | C | A | 417580 | 0.00310724 | 0.0279578 | ebi-a-GCST90000615 | 0.113012 | 2.31E-19 | 0.000156703 | 65.44612824 |
| 101 | rs8121940 | G | C | 417580 | 0.00247344 | -0.037716 | ebi-a-GCST90000615 | 0.195248 | 1.69E-52 | 0.000447023 | 186.7504691 |
| 102 | rs2229742 | C | G | 417580 | 0.00322012 | -0.0251367 | ebi-a-GCST90000615 | 0.103467 | 5.90E-15 | 0.000117223 | 48.95569032 |
| 103 | rs6003465 | C | T | 417580 | 0.0020906 | -0.0119188 | ebi-a-GCST90000615 | 0.331983 | 1.19E-08 | 6.30E-05 | 26.31257767 |
| 104 | rs2074735 | C | G | 417580 | 0.00400859 | 0.027808 | ebi-a-GCST90000615 | 0.064056 | 4.00E-12 | 9.27E-05 | 38.72193622 |
| 105 | rs115621755 | T | C | 417580 | 0.00209027 | -0.012627 | ebi-a-GCST90000615 | 0.327127 | 1.53E-09 | 7.02E-05 | 29.31216818 |

SNP, single nucleotide polymorphism; EA, effect allele; OA, other allele; EAF, effect allele frequency; SE, standard error.

**Supplementary Table S2** SNPs from GWAS on serum 25-hydroxyvitamin D levels and autoimmune thyroiditis

|  |  | | | **Exposure (Serum 25-Hydroxyvitamin D levels)** | | |  | **Outcome (Autoimmune thyroiditis)** | | | | |
| --- | --- | --- | --- | --- | --- | --- | --- | --- | --- | --- | --- | --- |
|  | **SNP** | **EA** | **OA** | **β** | **SE** | ***P* value** |  | **Case** | **Control** | **β** | **SE** | ***P* value** |
| 1 | rs1047891 | A | C | -0.0125534 | 0.00210766 | 2.58E-09 |  | 489 | 320,703 | -0.00966833 | 0.069012 | 0.888584 |
| 2 | rs10832277 | A | G | -0.075469 | 0.00204399 | 2.02E-298 |  | 489 | 320,703 | -0.0217588 | 0.0670156 | 0.745422 |
| 3 | rs10859995 | C | T | -0.0399464 | 0.00198939 | 1.11E-89 |  | 489 | 320,703 | -0.011001 | 0.0678618 | 0.87122 |
| 4 | rs10908469 | C | A | 0.0158902 | 0.00221124 | 6.67E-13 |  | 489 | 320,703 | -0.0458552 | 0.0711908 | 0.519501 |
| 5 | rs11076175 | G | A | 0.023978 | 0.00256672 | 9.46E-21 |  | 489 | 320,703 | -0.17412 | 0.0849056 | 0.0402912 |
| 6 | rs11182428 | C | T | -0.0123489 | 0.00196317 | 3.17E-10 |  | 489 | 320,703 | 0.00104792 | 0.0643355 | 0.987004 |
| 7 | rs11542462 | A | G | -0.0232523 | 0.00287287 | 5.79E-16 |  | 489 | 320,703 | -0.0603104 | 0.110187 | 0.584141 |
| 8 | rs117862422 | C | T | -0.0541519 | 0.00850362 | 1.91E-10 |  | 489 | 320,703 | 0.0598997 | 0.161779 | 0.711191 |
| 9 | rs12056768 | G | T | -0.021949 | 0.00199283 | 3.27E-28 |  | 489 | 320,703 | 0.0405181 | 0.064252 | 0.528293 |
| 10 | rs12123821 | T | C | 0.0772297 | 0.00460381 | 3.71E-63 |  | 489 | 320,703 | -0.0329475 | 0.166046 | 0.842713 |
| 11 | rs12317268 | G | A | -0.0206326 | 0.00274181 | 5.26E-14 |  | 489 | 320,703 | 0.0765265 | 0.0721142 | 0.288606 |
| 12 | rs12462826 | A | G | -0.0131168 | 0.00204832 | 1.52E-10 |  | 489 | 320,703 | -0.0270817 | 0.0681656 | 0.691153 |
| 13 | rs12949853 | A | G | 0.0144344 | 0.00253426 | 1.23E-08 |  | 489 | 320,703 | 0.00313449 | 0.0847214 | 0.970487 |
| 14 | rs13011615 | T | A | 0.0169587 | 0.00288288 | 4.04E-09 |  | 489 | 320,703 | -0.201485 | 0.0932226 | 0.0306697 |
| 15 | rs13284054 | C | T | 0.0185546 | 0.00308554 | 1.82E-09 |  | 489 | 320,703 | 0.0312754 | 0.102358 | 0.759948 |
| 16 | rs138072379 | T | C | 0.0542072 | 0.00720764 | 5.44E-14 |  | 489 | 320,703 | -0.0674571 | 0.226468 | 0.765806 |
| 17 | rs142004400 | C | A | -0.0332364 | 0.00537846 | 6.43E-10 |  | 489 | 320,703 | 0.0066883 | 0.223336 | 0.976109 |
| 18 | rs142158911 | A | G | 0.0266711 | 0.00309689 | 7.17E-18 |  | 489 | 320,703 | -0.0617089 | 0.105874 | 0.559994 |
| 19 | rs143069752 | A | T | 0.0224092 | 0.00392801 | 1.16E-08 |  | 489 | 320,703 | 0.211966 | 0.135061 | 0.116551 |
| 20 | rs143488652 | G | A | -0.0629543 | 0.00855038 | 1.80E-13 |  | 489 | 320,703 | -0.120249 | 0.683009 | 0.860249 |
| 21 | rs146128209 | G | A | -0.057293 | 0.00383087 | 1.43E-50 |  | 489 | 320,703 | -0.0745356 | 0.155994 | 0.632785 |
| 22 | rs1532085 | G | A | 0.0260168 | 0.0020142 | 3.62E-38 |  | 489 | 320,703 | -0.0356203 | 0.0649375 | 0.583327 |
| 23 | rs17144574 | C | T | -0.01526 | 0.00233815 | 6.73E-11 |  | 489 | 320,703 | -0.00939562 | 0.0771887 | 0.903118 |
| 24 | rs1800588 | T | C | -0.0329963 | 0.00238443 | 1.50E-43 |  | 489 | 320,703 | 0.00287427 | 0.0744806 | 0.969217 |
| 25 | rs182244780 | A | G | -0.356294 | 0.00864543 | 0 |  | 489 | 320,703 | -0.624353 | 0.593962 | 0.293182 |
| 26 | rs1858889 | C | A | 0.0111372 | 0.00196228 | 1.38E-08 |  | 489 | 320,703 | 0.00277723 | 0.0644212 | 0.965613 |
| 27 | rs189407772 | G | A | 0.0530974 | 0.00661678 | 1.02E-15 |  | 489 | 320,703 | -0.134428 | 0.450664 | 0.765482 |
| 28 | rs1894100 | T | G | -0.096459 | 0.00253888 | 0 |  | 489 | 320,703 | 0.132878 | 0.06719 | 0.0479689 |
| 29 | rs1966478 | C | T | -0.0123541 | 0.00213261 | 6.92E-09 |  | 489 | 320,703 | 0.0585848 | 0.0686464 | 0.393421 |
| 30 | rs2012736 | A | C | -0.0481921 | 0.00360843 | 1.10E-40 |  | 489 | 320,703 | 0.00537323 | 0.120587 | 0.964459 |
| 31 | rs2037511 | A | G | 0.0170377 | 0.00263857 | 1.07E-10 |  | 489 | 320,703 | 0.125566 | 0.0873871 | 0.150748 |
| 32 | rs2074735 | C | G | 0.027808 | 0.00400859 | 4.00E-12 |  | 489 | 320,703 | 0.0166574 | 0.092917 | 0.857724 |
| 33 | rs212100 | C | T | -0.065701 | 0.00264866 | 7.84E-136 |  | 489 | 320,703 | 0.0495621 | 0.0902505 | 0.582895 |
| 34 | rs2123930 | A | G | -0.014114 | 0.00219611 | 1.30E-10 |  | 489 | 320,703 | 0.15713 | 0.0693778 | 0.0235218 |
| 35 | rs2131925 | T | G | -0.0222318 | 0.00205242 | 2.43E-27 |  | 489 | 320,703 | 0.123272 | 0.072759 | 0.0902173 |
| 36 | rs2207132 | A | G | -0.0386454 | 0.00549169 | 1.96E-12 |  | 489 | 320,703 | -0.0420478 | 0.131718 | 0.749556 |
| 37 | rs2229742 | C | G | -0.0251367 | 0.00322012 | 5.90E-15 |  | 489 | 320,703 | 0.070752 | 0.10221 | 0.488798 |
| 38 | rs2297991 | C | T | 0.0120361 | 0.00218295 | 3.51E-08 |  | 489 | 320,703 | 0.0448852 | 0.0680971 | 0.509808 |
| 39 | rs2346264 | C | A | -0.0147004 | 0.00239864 | 8.86E-10 |  | 489 | 320,703 | 0.136456 | 0.0879749 | 0.120884 |
| 40 | rs2608984 | T | A | -0.0208623 | 0.00264074 | 2.79E-15 |  | 489 | 320,703 | 0.250035 | 0.0730516 | 0.000619998 |
| 41 | rs2642439 | G | A | -0.0145212 | 0.00211201 | 6.18E-12 |  | 489 | 320,703 | -0.0156426 | 0.0718038 | 0.827545 |
| 42 | rs2659007 | A | G | 0.0114099 | 0.00198953 | 9.75E-09 |  | 489 | 320,703 | -0.085715 | 0.0645673 | 0.184334 |
| 43 | rs2756119 | A | G | 0.0121881 | 0.0020411 | 2.35E-09 |  | 489 | 320,703 | 0.0891856 | 0.0662462 | 0.178214 |
| 44 | rs2847500 | A | G | -0.0221038 | 0.00298092 | 1.22E-13 |  | 489 | 320,703 | -0.0303181 | 0.090692 | 0.738156 |
| 45 | rs2952289 | T | C | 0.0178756 | 0.00245386 | 3.22E-13 |  | 489 | 320,703 | -0.0269226 | 0.0837475 | 0.747853 |
| 46 | rs34177108 | A | C | -0.0122879 | 0.00223709 | 3.96E-08 |  | 489 | 320,703 | -0.0291887 | 0.0801372 | 0.715683 |
| 47 | rs34186890 | G | A | -0.0149926 | 0.00224148 | 2.25E-11 |  | 489 | 320,703 | -0.0754768 | 0.0742792 | 0.309572 |
| 48 | rs34284484 | G | T | -0.0123129 | 0.002169 | 1.37E-08 |  | 489 | 320,703 | -0.0111392 | 0.0724435 | 0.877796 |
| 49 | rs34726834 | T | C | 0.0149657 | 0.00227206 | 4.49E-11 |  | 489 | 320,703 | 0.0274461 | 0.0704671 | 0.696915 |
| 50 | rs35408430 | T | C | -0.0213322 | 0.00206739 | 5.82E-25 |  | 489 | 320,703 | 0.00176664 | 0.0669449 | 0.978947 |
| 51 | rs3814995 | T | C | -0.0126428 | 0.00211677 | 2.33E-09 |  | 489 | 320,703 | -0.0664325 | 0.067649 | 0.326091 |
| 52 | rs3890624 | G | A | 0.0112736 | 0.00203846 | 3.19E-08 |  | 489 | 320,703 | -0.0682932 | 0.0675997 | 0.312371 |
| 53 | rs4121823 | A | T | -0.0185043 | 0.00273512 | 1.33E-11 |  | 489 | 320,703 | -0.0909022 | 0.0866763 | 0.294291 |
| 54 | rs4306928 | C | T | 0.0250598 | 0.00263067 | 1.63E-21 |  | 489 | 320,703 | 0.037285 | 0.086066 | 0.664858 |
| 55 | rs4364259 | A | G | 0.0165067 | 0.0024674 | 2.23E-11 |  | 489 | 320,703 | -0.0635213 | 0.0777906 | 0.414175 |
| 56 | rs4418728 | T | G | 0.0121203 | 0.00196937 | 7.54E-10 |  | 489 | 320,703 | -0.0439676 | 0.0641983 | 0.493425 |
| 57 | rs4694423 | A | C | -0.100874 | 0.00199111 | 0 |  | 489 | 320,703 | 0.122305 | 0.0649821 | 0.0598191 |
| 58 | rs512083 | C | T | 0.0122018 | 0.00197554 | 6.56E-10 |  | 489 | 320,703 | -0.00145922 | 0.0642572 | 0.981882 |
| 59 | rs57459725 | G | C | -0.0176615 | 0.00289594 | 1.07E-09 |  | 489 | 320,703 | 0.0972417 | 0.0853387 | 0.254503 |
| 60 | rs57938057 | C | T | -0.0787942 | 0.00745585 | 4.19E-26 |  | 489 | 320,703 | -0.102628 | 0.150645 | 0.49571 |
| 61 | rs58387006 | C | A | -0.0137174 | 0.0023619 | 6.33E-09 |  | 489 | 320,703 | 0.02502 | 0.0761079 | 0.742349 |
| 62 | rs6003465 | C | T | -0.0119188 | 0.0020906 | 1.19E-08 |  | 489 | 320,703 | -0.0782363 | 0.0720693 | 0.277669 |
| 63 | rs61815559 | T | A | 0.0845633 | 0.00564426 | 9.60E-51 |  | 489 | 320,703 | 0.0358533 | 0.362477 | 0.921208 |
| 64 | rs62012766 | C | T | -0.0181582 | 0.00269511 | 1.61E-11 |  | 489 | 320,703 | 0.0248254 | 0.0764427 | 0.745365 |
| 65 | rs62115743 | T | C | 0.0274464 | 0.00361625 | 3.21E-14 |  | 489 | 320,703 | 0.0076749 | 0.10762 | 0.943147 |
| 66 | rs6438900 | G | C | 0.0133459 | 0.00225847 | 3.44E-09 |  | 489 | 320,703 | 0.0741916 | 0.0701834 | 0.290461 |
| 67 | rs6547409 | T | C | 0.0279437 | 0.00452445 | 6.57E-10 |  | 489 | 320,703 | -0.0235514 | 0.121092 | 0.84579 |
| 68 | rs6671730 | A | G | -0.015115 | 0.00197958 | 2.25E-14 |  | 489 | 320,703 | 0.044672 | 0.0641338 | 0.486088 |
| 69 | rs6723486 | T | C | -0.0111173 | 0.00202854 | 4.24E-08 |  | 489 | 320,703 | 0.12203 | 0.0645277 | 0.0586084 |
| 70 | rs6724965 | G | A | -0.0165826 | 0.00259881 | 1.76E-10 |  | 489 | 320,703 | -0.116637 | 0.0785986 | 0.137821 |
| 71 | rs6834488 | T | C | -0.0146317 | 0.00199028 | 1.96E-13 |  | 489 | 320,703 | 0.0590788 | 0.0681481 | 0.385986 |
| 72 | rs71601787 | A | G | 0.0419281 | 0.00210912 | 6.13E-88 |  | 489 | 320,703 | 0.0314993 | 0.0705556 | 0.655276 |
| 73 | rs72834856 | G | T | -0.0235978 | 0.00379173 | 4.86E-10 |  | 489 | 320,703 | -0.0013004 | 0.133078 | 0.992203 |
| 74 | rs72997688 | G | A | 0.0243759 | 0.00422905 | 8.22E-09 |  | 489 | 320,703 | -0.0673584 | 0.126169 | 0.593429 |
| 75 | rs733454 | T | C | 0.0195549 | 0.00328005 | 2.50E-09 |  | 489 | 320,703 | 0.156772 | 0.14049 | 0.264465 |
| 76 | rs73413596 | C | T | 0.0237483 | 0.00376674 | 2.89E-10 |  | 489 | 320,703 | -0.134076 | 0.13621 | 0.324953 |
| 77 | rs7439366 | C | T | -0.0315734 | 0.00196647 | 5.20E-58 |  | 489 | 320,703 | -0.0564304 | 0.0647114 | 0.383191 |
| 78 | rs7522116 | T | C | -0.0126086 | 0.00199431 | 2.58E-10 |  | 489 | 320,703 | -0.088651 | 0.0657046 | 0.177261 |
| 79 | rs7569755 | A | G | 0.0132378 | 0.00217782 | 1.21E-09 |  | 489 | 320,703 | 0.0366623 | 0.0777217 | 0.637131 |
| 80 | rs7657132 | G | A | -0.0140781 | 0.00213658 | 4.43E-11 |  | 489 | 320,703 | 0.0661643 | 0.06925 | 0.339355 |
| 81 | rs77924615 | A | G | -0.0144881 | 0.00251244 | 8.09E-09 |  | 489 | 320,703 | 0.128511 | 0.0770051 | 0.0951459 |
| 82 | rs78649910 | A | T | -0.0193309 | 0.00320205 | 1.57E-09 |  | 489 | 320,703 | 0.06576 | 0.0985168 | 0.504452 |
| 83 | rs79598313 | T | C | -0.0370228 | 0.00648419 | 1.13E-08 |  | 489 | 320,703 | 0.0617234 | 0.177498 | 0.728035 |
| 84 | rs79687284 | C | G | -0.0295438 | 0.00537042 | 3.77E-08 |  | 489 | 320,703 | 0.225224 | 0.239649 | 0.347316 |
| 85 | rs8018720 | C | G | -0.0383637 | 0.0025687 | 1.95E-50 |  | 489 | 320,703 | -0.0714015 | 0.0897403 | 0.426238 |
| 86 | rs804281 | G | A | 0.0157516 | 0.00199025 | 2.48E-15 |  | 489 | 320,703 | -0.0662457 | 0.0695126 | 0.340589 |
| 87 | rs8063565 | C | G | 0.0123302 | 0.00221722 | 2.68E-08 |  | 489 | 320,703 | 0.0408735 | 0.0706262 | 0.562771 |
| 88 | rs8091117 | A | C | -0.02514 | 0.0039672 | 2.34E-10 |  | 489 | 320,703 | 0.16457 | 0.119217 | 0.167454 |
| 89 | rs8107974 | T | A | 0.0386164 | 0.00369585 | 1.49E-25 |  | 489 | 320,703 | 0.0167213 | 0.12881 | 0.896714 |
| 90 | rs8121940 | G | C | -0.037716 | 0.00247344 | 1.69E-52 |  | 489 | 320,703 | -0.0581699 | 0.0760996 | 0.444633 |
| 91 | rs8181687 | A | G | 0.0116958 | 0.00198761 | 4.00E-09 |  | 489 | 320,703 | -0.0720672 | 0.0649672 | 0.267307 |
| 92 | rs9409266 | A | G | -0.0184021 | 0.00283662 | 8.74E-11 |  | 489 | 320,703 | 0.106301 | 0.0858468 | 0.215619 |
| 93 | rs9467550 | G | A | -0.0188955 | 0.00319549 | 3.36E-09 |  | 489 | 320,703 | 0.014027 | 0.102053 | 0.890677 |
| 94 | rs9476310 | T | C | 0.0117234 | 0.00197015 | 2.67E-09 |  | 489 | 320,703 | -0.00164771 | 0.0644056 | 0.97959 |
| 95 | rs964184 | C | G | 0.0431884 | 0.00289883 | 3.37E-50 |  | 489 | 320,703 | -0.143497 | 0.0911049 | 0.11524 |

SNP, single nucleotide polymorphism; EA, effect allele; OA, other allele; SE, standard error.

**Supplementary Table S3** SNPs from GWAS on serum 25-hydroxyvitamin D levels and autoimmune hyperthyroidism

|  |  | | | **Exposure (Serum 25-Hydroxyvitamin D levels)** | | |  | **Outcome (Autoimmune hyperthyroidism)** | | | | |
| --- | --- | --- | --- | --- | --- | --- | --- | --- | --- | --- | --- | --- |
|  | **SNP** | **EA** | **OA** | **β** | **SE** | ***P* value** |  | **Case** | **Control** | **β** | **SE** | ***P* value** |
| 1 | rs1047891 | A | C | -0.0125534 | 0.00210766 | 2.58E-09 |  | 1,828 | 279,855 | 0.000524579 | 0.0357968 | 0.988308 |
| 2 | rs10832277 | A | G | -0.075469 | 0.00204399 | 2.02E-298 |  | 1,828 | 279,855 | 0.0202587 | 0.0348189 | 0.56068 |
| 3 | rs10859995 | C | T | -0.0399464 | 0.00198939 | 1.11E-89 |  | 1,828 | 279,855 | -0.0571815 | 0.0352218 | 0.10449 |
| 4 | rs10908469 | C | A | 0.0158902 | 0.00221124 | 6.67E-13 |  | 1,828 | 279,855 | -0.0268342 | 0.0370376 | 0.468751 |
| 5 | rs11076175 | G | A | 0.023978 | 0.00256672 | 9.46E-21 |  | 1,828 | 279,855 | 0.0470385 | 0.0441679 | 0.286879 |
| 6 | rs11182428 | C | T | -0.0123489 | 0.00196317 | 3.17E-10 |  | 1,828 | 279,855 | 0.049226 | 0.0333668 | 0.140133 |
| 7 | rs11542462 | A | G | -0.0232523 | 0.00287287 | 5.79E-16 |  | 1,828 | 279,855 | 0.0416064 | 0.0572674 | 0.467515 |
| 8 | rs117862422 | C | T | -0.0541519 | 0.00850362 | 1.91E-10 |  | 1,828 | 279,855 | 0.0354131 | 0.0847442 | 0.676033 |
| 9 | rs12056768 | G | T | -0.021949 | 0.00199283 | 3.27E-28 |  | 1,828 | 279,855 | 0.00327685 | 0.0333794 | 0.921797 |
| 10 | rs12123821 | T | C | 0.0772297 | 0.00460381 | 3.71E-63 |  | 1,828 | 279,855 | -0.0584827 | 0.0864482 | 0.49872 |
| 11 | rs12317268 | G | A | -0.0206326 | 0.00274181 | 5.26E-14 |  | 1,828 | 279,855 | -0.0530433 | 0.0373846 | 0.155941 |
| 12 | rs12462826 | A | G | -0.0131168 | 0.00204832 | 1.52E-10 |  | 1,828 | 279,855 | 0.0338905 | 0.0354397 | 0.338928 |
| 13 | rs12949853 | A | G | 0.0144344 | 0.00253426 | 1.23E-08 |  | 1,828 | 279,855 | -0.0174287 | 0.0440407 | 0.692296 |
| 14 | rs13011615 | T | A | 0.0169587 | 0.00288288 | 4.04E-09 |  | 1,828 | 279,855 | -0.00359477 | 0.0482517 | 0.940612 |
| 15 | rs13284054 | C | T | 0.0185546 | 0.00308554 | 1.82E-09 |  | 1,828 | 279,855 | -0.079813 | 0.0537343 | 0.137457 |
| 16 | rs138072379 | T | C | 0.0542072 | 0.00720764 | 5.44E-14 |  | 1,828 | 279,855 | 0.117277 | 0.116717 | 0.314995 |
| 17 | rs142004400 | C | A | -0.0332364 | 0.00537846 | 6.43E-10 |  | 1,828 | 279,855 | -0.17496 | 0.119763 | 0.144049 |
| 18 | rs142158911 | A | G | 0.0266711 | 0.00309689 | 7.17E-18 |  | 1,828 | 279,855 | -0.0175088 | 0.0551052 | 0.750687 |
| 19 | rs143069752 | A | T | 0.0224092 | 0.00392801 | 1.16E-08 |  | 1,828 | 279,855 | 0.0881303 | 0.0709315 | 0.214063 |
| 20 | rs143488652 | G | A | -0.0629543 | 0.00855038 | 1.80E-13 |  | 1,828 | 279,855 | 0.0385413 | 0.349551 | 0.912203 |
| 21 | rs146128209 | G | A | -0.057293 | 0.00383087 | 1.43E-50 |  | 1,828 | 279,855 | 0.00687646 | 0.0823744 | 0.933471 |
| 22 | rs1532085 | G | A | 0.0260168 | 0.0020142 | 3.62E-38 |  | 1,828 | 279,855 | 0.01224 | 0.0337955 | 0.717218 |
| 23 | rs17144574 | C | T | -0.01526 | 0.00233815 | 6.73E-11 |  | 1,828 | 279,855 | 0.0114171 | 0.0402402 | 0.776623 |
| 24 | rs1800588 | T | C | -0.0329963 | 0.00238443 | 1.50E-43 |  | 1,828 | 279,855 | 0.00121917 | 0.0386187 | 0.974815 |
| 25 | rs182244780 | A | G | -0.356294 | 0.00864543 | 0 |  | 1,828 | 279,855 | -0.142086 | 0.311033 | 0.6478 |
| 26 | rs1858889 | C | A | 0.0111372 | 0.00196228 | 1.38E-08 |  | 1,828 | 279,855 | -0.00323808 | 0.0333937 | 0.922753 |
| 27 | rs189407772 | G | A | 0.0530974 | 0.00661678 | 1.02E-15 |  | 1,828 | 279,855 | 0.305847 | 0.234836 | 0.192784 |
| 28 | rs1894100 | T | G | -0.096459 | 0.00253888 | 0 |  | 1,828 | 279,855 | -0.024967 | 0.0349465 | 0.474958 |
| 29 | rs1966478 | C | T | -0.0123541 | 0.00213261 | 6.92E-09 |  | 1,828 | 279,855 | 0.0587708 | 0.0357203 | 0.0999056 |
| 30 | rs2012736 | A | C | -0.0481921 | 0.00360843 | 1.10E-40 |  | 1,828 | 279,855 | 0.113911 | 0.0630438 | 0.0707864 |
| 31 | rs2037511 | A | G | 0.0170377 | 0.00263857 | 1.07E-10 |  | 1,828 | 279,855 | 0.0194683 | 0.0453698 | 0.667849 |
| 32 | rs2074735 | C | G | 0.027808 | 0.00400859 | 4.00E-12 |  | 1,828 | 279,855 | 0.0288098 | 0.0480987 | 0.549192 |
| 33 | rs212100 | C | T | -0.065701 | 0.00264866 | 7.84E-136 |  | 1,828 | 279,855 | -0.00623316 | 0.046846 | 0.894149 |
| 34 | rs2123930 | A | G | -0.014114 | 0.00219611 | 1.30E-10 |  | 1,828 | 279,855 | -0.0181862 | 0.0358999 | 0.612449 |
| 35 | rs2131925 | T | G | -0.0222318 | 0.00205242 | 2.43E-27 |  | 1,828 | 279,855 | 0.0520276 | 0.0379054 | 0.169888 |
| 36 | rs2207132 | A | G | -0.0386454 | 0.00549169 | 1.96E-12 |  | 1,828 | 279,855 | 0.171828 | 0.0678649 | 0.0113441 |
| 37 | rs2229742 | C | G | -0.0251367 | 0.00322012 | 5.90E-15 |  | 1,828 | 279,855 | 0.0728994 | 0.0530079 | 0.169052 |
| 38 | rs2297991 | C | T | 0.0120361 | 0.00218295 | 3.51E-08 |  | 1,828 | 279,855 | -0.0263186 | 0.0353402 | 0.456439 |
| 39 | rs2346264 | C | A | -0.0147004 | 0.00239864 | 8.86E-10 |  | 1,828 | 279,855 | 0.0736617 | 0.0458402 | 0.108071 |
| 40 | rs2608984 | T | A | -0.0208623 | 0.00264074 | 2.79E-15 |  | 1,828 | 279,855 | 0.0496224 | 0.0407336 | 0.223142 |
| 41 | rs2642439 | G | A | -0.0145212 | 0.00211201 | 6.18E-12 |  | 1,828 | 279,855 | 0.0653861 | 0.0373129 | 0.0797095 |
| 42 | rs2659007 | A | G | 0.0114099 | 0.00198953 | 9.75E-09 |  | 1,828 | 279,855 | 0.00294119 | 0.0335384 | 0.930118 |
| 43 | rs2756119 | A | G | 0.0121881 | 0.0020411 | 2.35E-09 |  | 1,828 | 279,855 | -0.0246597 | 0.0343415 | 0.472712 |
| 44 | rs2847500 | A | G | -0.0221038 | 0.00298092 | 1.22E-13 |  | 1,828 | 279,855 | -0.00297449 | 0.047442 | 0.950007 |
| 45 | rs2952289 | T | C | 0.0178756 | 0.00245386 | 3.22E-13 |  | 1,828 | 279,855 | 0.0440551 | 0.0436716 | 0.313079 |
| 46 | rs34177108 | A | C | -0.0122879 | 0.00223709 | 3.96E-08 |  | 1,828 | 279,855 | -0.02187 | 0.041798 | 0.600813 |
| 47 | rs34186890 | G | A | -0.0149926 | 0.00224148 | 2.25E-11 |  | 1,828 | 279,855 | -0.00315692 | 0.0387027 | 0.93499 |
| 48 | rs34284484 | G | T | -0.0123129 | 0.002169 | 1.37E-08 |  | 1,828 | 279,855 | -0.0466771 | 0.0376506 | 0.215071 |
| 49 | rs34726834 | T | C | 0.0149657 | 0.00227206 | 4.49E-11 |  | 1,828 | 279,855 | 0.00747368 | 0.0364268 | 0.83744 |
| 50 | rs35408430 | T | C | -0.0213322 | 0.00206739 | 5.82E-25 |  | 1,828 | 279,855 | -0.038527 | 0.03477 | 0.267839 |
| 51 | rs3814995 | T | C | -0.0126428 | 0.00211677 | 2.33E-09 |  | 1,828 | 279,855 | -0.0784829 | 0.0350858 | 0.0252936 |
| 52 | rs3890624 | G | A | 0.0112736 | 0.00203846 | 3.19E-08 |  | 1,828 | 279,855 | -0.0446322 | 0.0352201 | 0.205071 |
| 53 | rs4121823 | A | T | -0.0185043 | 0.00273512 | 1.33E-11 |  | 1,828 | 279,855 | 0.00737441 | 0.0453505 | 0.870826 |
| 54 | rs4306928 | C | T | 0.0250598 | 0.00263067 | 1.63E-21 |  | 1,828 | 279,855 | -0.00506426 | 0.0450067 | 0.910409 |
| 55 | rs4364259 | A | G | 0.0165067 | 0.0024674 | 2.23E-11 |  | 1,828 | 279,855 | 0.0210343 | 0.0404041 | 0.602647 |
| 56 | rs4418728 | T | G | 0.0121203 | 0.00196937 | 7.54E-10 |  | 1,828 | 279,855 | -0.0306193 | 0.0333561 | 0.358644 |
| 57 | rs4694423 | A | C | -0.100874 | 0.00199111 | 0 |  | 1,828 | 279,855 | -0.0173517 | 0.0337216 | 0.606862 |
| 58 | rs512083 | C | T | 0.0122018 | 0.00197554 | 6.56E-10 |  | 1,828 | 279,855 | -0.017586 | 0.0333262 | 0.597713 |
| 59 | rs57459725 | G | C | -0.0176615 | 0.00289594 | 1.07E-09 |  | 1,828 | 279,855 | 0.0138383 | 0.0442472 | 0.75447 |
| 60 | rs57938057 | C | T | -0.0787942 | 0.00745585 | 4.19E-26 |  | 1,828 | 279,855 | -0.0290716 | 0.0771723 | 0.706389 |
| 61 | rs58387006 | C | A | -0.0137174 | 0.0023619 | 6.33E-09 |  | 1,828 | 279,855 | 0.0418433 | 0.0395698 | 0.290303 |
| 62 | rs6003465 | C | T | -0.0119188 | 0.0020906 | 1.19E-08 |  | 1,828 | 279,855 | -0.0052126 | 0.0375104 | 0.889479 |
| 63 | rs61815559 | T | A | 0.0845633 | 0.00564426 | 9.60E-51 |  | 1,828 | 279,855 | -0.160786 | 0.184012 | 0.382238 |
| 64 | rs62012766 | C | T | -0.0181582 | 0.00269511 | 1.61E-11 |  | 1,828 | 279,855 | 0.0283246 | 0.0397571 | 0.476192 |
| 65 | rs62115743 | T | C | 0.0274464 | 0.00361625 | 3.21E-14 |  | 1,828 | 279,855 | -0.0311262 | 0.055575 | 0.575428 |
| 66 | rs6438900 | G | C | 0.0133459 | 0.00225847 | 3.44E-09 |  | 1,828 | 279,855 | 0.0252632 | 0.0362662 | 0.486049 |
| 67 | rs6547409 | T | C | 0.0279437 | 0.00452445 | 6.57E-10 |  | 1,828 | 279,855 | 0.0414143 | 0.0622093 | 0.505587 |
| 68 | rs6671730 | A | G | -0.015115 | 0.00197958 | 2.25E-14 |  | 1,828 | 279,855 | -0.0231228 | 0.0332991 | 0.487434 |
| 69 | rs6723486 | T | C | -0.0111173 | 0.00202854 | 4.24E-08 |  | 1,828 | 279,855 | -0.00572194 | 0.0334784 | 0.864291 |
| 70 | rs6724965 | G | A | -0.0165826 | 0.00259881 | 1.76E-10 |  | 1,828 | 279,855 | 0.0555463 | 0.0405451 | 0.17069 |
| 71 | rs6834488 | T | C | -0.0146317 | 0.00199028 | 1.96E-13 |  | 1,828 | 279,855 | 0.00810777 | 0.035609 | 0.819889 |
| 72 | rs71601787 | A | G | 0.0419281 | 0.00210912 | 6.13E-88 |  | 1,828 | 279,855 | -0.0336493 | 0.0366646 | 0.358746 |
| 73 | rs72834856 | G | T | -0.0235978 | 0.00379173 | 4.86E-10 |  | 1,828 | 279,855 | -0.136516 | 0.0685017 | 0.0462732 |
| 74 | rs72997688 | G | A | 0.0243759 | 0.00422905 | 8.22E-09 |  | 1,828 | 279,855 | 0.0692339 | 0.0651711 | 0.288081 |
| 75 | rs733454 | T | C | 0.0195549 | 0.00328005 | 2.50E-09 |  | 1,828 | 279,855 | 0.0239425 | 0.0725361 | 0.741343 |
| 76 | rs73413596 | C | T | 0.0237483 | 0.00376674 | 2.89E-10 |  | 1,828 | 279,855 | 0.0913763 | 0.0712298 | 0.199549 |
| 77 | rs7439366 | C | T | -0.0315734 | 0.00196647 | 5.20E-58 |  | 1,828 | 279,855 | -0.0502356 | 0.0336243 | 0.135168 |
| 78 | rs7522116 | T | C | -0.0126086 | 0.00199431 | 2.58E-10 |  | 1,828 | 279,855 | 0.0507283 | 0.0340855 | 0.136681 |
| 79 | rs7569755 | A | G | 0.0132378 | 0.00217782 | 1.21E-09 |  | 1,828 | 279,855 | -0.0145458 | 0.0405104 | 0.719548 |
| 80 | rs7657132 | G | A | -0.0140781 | 0.00213658 | 4.43E-11 |  | 1,828 | 279,855 | 0.0523985 | 0.0360814 | 0.146437 |
| 81 | rs77924615 | A | G | -0.0144881 | 0.00251244 | 8.09E-09 |  | 1,828 | 279,855 | 0.0558819 | 0.0401672 | 0.164155 |
| 82 | rs78649910 | A | T | -0.0193309 | 0.00320205 | 1.57E-09 |  | 1,828 | 279,855 | -0.0293682 | 0.0510159 | 0.564839 |
| 83 | rs79598313 | T | C | -0.0370228 | 0.00648419 | 1.13E-08 |  | 1,828 | 279,855 | -0.138986 | 0.0914671 | 0.128631 |
| 84 | rs79687284 | C | G | -0.0295438 | 0.00537042 | 3.77E-08 |  | 1,828 | 279,855 | -0.0458897 | 0.126885 | 0.717603 |
| 85 | rs8018720 | C | G | -0.0383637 | 0.0025687 | 1.95E-50 |  | 1,828 | 279,855 | 0.0806059 | 0.0465287 | 0.0832032 |
| 86 | rs804281 | G | A | 0.0157516 | 0.00199025 | 2.48E-15 |  | 1,828 | 279,855 | -0.029292 | 0.0361158 | 0.417333 |
| 87 | rs8063565 | C | G | 0.0123302 | 0.00221722 | 2.68E-08 |  | 1,828 | 279,855 | -0.000134858 | 0.0367579 | 0.997073 |
| 88 | rs8091117 | A | C | -0.02514 | 0.0039672 | 2.34E-10 |  | 1,828 | 279,855 | 0.131525 | 0.0621448 | 0.0343084 |
| 89 | rs8107974 | T | A | 0.0386164 | 0.00369585 | 1.49E-25 |  | 1,828 | 279,855 | -0.0464132 | 0.067715 | 0.49308 |
| 90 | rs8121940 | G | C | -0.037716 | 0.00247344 | 1.69E-52 |  | 1,828 | 279,855 | -0.00245739 | 0.0393702 | 0.95023 |
| 91 | rs8181687 | A | G | 0.0116958 | 0.00198761 | 4.00E-09 |  | 1,828 | 279,855 | -0.0284807 | 0.0337424 | 0.398636 |
| 92 | rs9409266 | A | G | -0.0184021 | 0.00283662 | 8.74E-11 |  | 1,828 | 279,855 | -0.00976481 | 0.0444102 | 0.825967 |
| 93 | rs9467550 | G | A | -0.0188955 | 0.00319549 | 3.36E-09 |  | 1,828 | 279,855 | -0.148059 | 0.054572 | 0.00666577 |
| 94 | rs9476310 | T | C | 0.0117234 | 0.00197015 | 2.67E-09 |  | 1,828 | 279,855 | 0.0100724 | 0.0334386 | 0.763247 |
| 95 | rs964184 | C | G | 0.0431884 | 0.00289883 | 3.37E-50 |  | 1,828 | 279,855 | -0.0646063 | 0.0472946 | 0.171927 |

SNP, single nucleotide polymorphism; EA, effect allele; OA, other allele; SE, standard error.

**Supplementary Table S4** SNPs from GWAS on serum 25-hydroxyvitamin D levels and Graves disease

|  |  | | | **Exposure (Serum 25-Hydroxyvitamin D levels)** | | |  | **Outcome (Graves disease)** | | | | |
| --- | --- | --- | --- | --- | --- | --- | --- | --- | --- | --- | --- | --- |
|  | **SNP** | **EA** | **OA** | **β** | **SE** | ***P* value** |  | **Case** | **Control** | **β** | **SE** | ***P* value** |
| 1 | rs1047891 | A | C | -0.0125534 | 0.00210766 | 2.58E-09 |  | 2,836 | 374,441 | 0.00197037 | 0.0287522 | 0.945364 |
| 2 | rs10832277 | A | G | -0.075469 | 0.00204399 | 2.02E-298 |  | 2,836 | 374,441 | 0.0317058 | 0.0279373 | 0.256421 |
| 3 | rs10859995 | C | T | -0.0399464 | 0.00198939 | 1.11E-89 |  | 2,836 | 374,441 | 0.0259286 | 0.0282771 | 0.35917 |
| 4 | rs10908469 | C | A | 0.0158902 | 0.00221124 | 6.67E-13 |  | 2,836 | 374,441 | 0.00234822 | 0.0297847 | 0.93716 |
| 5 | rs11076175 | G | A | 0.023978 | 0.00256672 | 9.46E-21 |  | 2,836 | 374,441 | -0.0141383 | 0.0353767 | 0.689414 |
| 6 | rs11182428 | C | T | -0.0123489 | 0.00196317 | 3.17E-10 |  | 2,836 | 374,441 | 0.046213 | 0.0268194 | 0.0848672 |
| 7 | rs11542462 | A | G | -0.0232523 | 0.00287287 | 5.79E-16 |  | 2,836 | 374,441 | -0.044247 | 0.0461032 | 0.337187 |
| 8 | rs117862422 | C | T | -0.0541519 | 0.00850362 | 1.91E-10 |  | 2,836 | 374,441 | 0.0818645 | 0.0680598 | 0.229041 |
| 9 | rs12056768 | G | T | -0.021949 | 0.00199283 | 3.27E-28 |  | 2,836 | 374,441 | -0.029384 | 0.0268009 | 0.272912 |
| 10 | rs12123821 | T | C | 0.0772297 | 0.00460381 | 3.71E-63 |  | 2,836 | 374,441 | -0.167033 | 0.06972 | 0.0165856 |
| 11 | rs12317268 | G | A | -0.0206326 | 0.00274181 | 5.26E-14 |  | 2,836 | 374,441 | -0.0225934 | 0.0300471 | 0.452091 |
| 12 | rs12462826 | A | G | -0.0131168 | 0.00204832 | 1.52E-10 |  | 2,836 | 374,441 | 0.00434571 | 0.0284341 | 0.878529 |
| 13 | rs12949853 | A | G | 0.0144344 | 0.00253426 | 1.23E-08 |  | 2,836 | 374,441 | -0.0436556 | 0.0353396 | 0.216713 |
| 14 | rs13011615 | T | A | 0.0169587 | 0.00288288 | 4.04E-09 |  | 2,836 | 374,441 | 0.0150457 | 0.0387984 | 0.69817 |
| 15 | rs13284054 | C | T | 0.0185546 | 0.00308554 | 1.82E-09 |  | 2,836 | 374,441 | -0.0457428 | 0.0432023 | 0.289689 |
| 16 | rs138072379 | T | C | 0.0542072 | 0.00720764 | 5.44E-14 |  | 2,836 | 374,441 | 0.00198577 | 0.0943711 | 0.983212 |
| 17 | rs142004400 | C | A | -0.0332364 | 0.00537846 | 6.43E-10 |  | 2,836 | 374,441 | -0.243667 | 0.0956257 | 0.01083 |
| 18 | rs142158911 | A | G | 0.0266711 | 0.00309689 | 7.17E-18 |  | 2,836 | 374,441 | 0.0400189 | 0.0444679 | 0.368147 |
| 19 | rs143069752 | A | T | 0.0224092 | 0.00392801 | 1.16E-08 |  | 2,836 | 374,441 | 0.0510112 | 0.056859 | 0.369637 |
| 20 | rs143488652 | G | A | -0.0629543 | 0.00855038 | 1.80E-13 |  | 2,836 | 374,441 | -0.0262559 | 0.281204 | 0.92561 |
| 21 | rs146128209 | G | A | -0.057293 | 0.00383087 | 1.43E-50 |  | 2,836 | 374,441 | -0.0506554 | 0.066055 | 0.44316 |
| 22 | rs1532085 | G | A | 0.0260168 | 0.0020142 | 3.62E-38 |  | 2,836 | 374,441 | -0.0383858 | 0.0271418 | 0.157282 |
| 23 | rs17144574 | C | T | -0.01526 | 0.00233815 | 6.73E-11 |  | 2,836 | 374,441 | 0.0272421 | 0.0322232 | 0.397877 |
| 24 | rs1800588 | T | C | -0.0329963 | 0.00238443 | 1.50E-43 |  | 2,836 | 374,441 | 0.0211251 | 0.0310271 | 0.49596 |
| 25 | rs182244780 | A | G | -0.356294 | 0.00864543 | 0 |  | 2,836 | 374,441 | 0.0925258 | 0.249777 | 0.71106 |
| 26 | rs1858889 | C | A | 0.0111372 | 0.00196228 | 1.38E-08 |  | 2,836 | 374,441 | 0.0349424 | 0.0268587 | 0.193268 |
| 27 | rs189407772 | G | A | 0.0530974 | 0.00661678 | 1.02E-15 |  | 2,836 | 374,441 | 0.00329322 | 0.190515 | 0.986209 |
| 28 | rs1894100 | T | G | -0.096459 | 0.00253888 | 0 |  | 2,836 | 374,441 | 0.0232522 | 0.0280703 | 0.407469 |
| 29 | rs1966478 | C | T | -0.0123541 | 0.00213261 | 6.92E-09 |  | 2,836 | 374,441 | 0.0385431 | 0.0286783 | 0.178953 |
| 30 | rs2012736 | A | C | -0.0481921 | 0.00360843 | 1.10E-40 |  | 2,836 | 374,441 | 0.124506 | 0.0486011 | 0.0104133 |
| 31 | rs2037511 | A | G | 0.0170377 | 0.00263857 | 1.07E-10 |  | 2,836 | 374,441 | 0.00395345 | 0.0365799 | 0.913935 |
| 32 | rs2074735 | C | G | 0.027808 | 0.00400859 | 4.00E-12 |  | 2,836 | 374,441 | -0.00273972 | 0.0387131 | 0.943581 |
| 33 | rs212100 | C | T | -0.065701 | 0.00264866 | 7.84E-136 |  | 2,836 | 374,441 | -0.0237875 | 0.0376496 | 0.527508 |
| 34 | rs2123930 | A | G | -0.014114 | 0.00219611 | 1.30E-10 |  | 2,836 | 374,441 | 0.0155014 | 0.0289009 | 0.591707 |
| 35 | rs2131925 | T | G | -0.0222318 | 0.00205242 | 2.43E-27 |  | 2,836 | 374,441 | 0.0194378 | 0.0303774 | 0.522253 |
| 36 | rs2207132 | A | G | -0.0386454 | 0.00549169 | 1.96E-12 |  | 2,836 | 374,441 | 0.0929456 | 0.0541888 | 0.0863058 |
| 37 | rs2229742 | C | G | -0.0251367 | 0.00322012 | 5.90E-15 |  | 2,836 | 374,441 | 0.0607239 | 0.0424645 | 0.152719 |
| 38 | rs2297991 | C | T | 0.0120361 | 0.00218295 | 3.51E-08 |  | 2,836 | 374,441 | -0.0756508 | 0.0280311 | 0.00695873 |
| 39 | rs2346264 | C | A | -0.0147004 | 0.00239864 | 8.86E-10 |  | 2,836 | 374,441 | 0.0667669 | 0.0367717 | 0.0694145 |
| 40 | rs2608984 | T | A | -0.0208623 | 0.00264074 | 2.79E-15 |  | 2,836 | 374,441 | -0.00641507 | 0.0326977 | 0.844459 |
| 41 | rs2642439 | G | A | -0.0145212 | 0.00211201 | 6.18E-12 |  | 2,836 | 374,441 | 0.0463294 | 0.0299476 | 0.121859 |
| 42 | rs2659007 | A | G | 0.0114099 | 0.00198953 | 9.75E-09 |  | 2,836 | 374,441 | 0.0331958 | 0.0269607 | 0.218224 |
| 43 | rs2756119 | A | G | 0.0121881 | 0.0020411 | 2.35E-09 |  | 2,836 | 374,441 | 0.00801641 | 0.027573 | 0.771255 |
| 44 | rs2847500 | A | G | -0.0221038 | 0.00298092 | 1.22E-13 |  | 2,836 | 374,441 | -0.00767443 | 0.0380636 | 0.840213 |
| 45 | rs2952289 | T | C | 0.0178756 | 0.00245386 | 3.22E-13 |  | 2,836 | 374,441 | 0.0421095 | 0.0350748 | 0.229921 |
| 46 | rs34177108 | A | C | -0.0122879 | 0.00223709 | 3.96E-08 |  | 2,836 | 374,441 | -0.0431371 | 0.0336196 | 0.19946 |
| 47 | rs34186890 | G | A | -0.0149926 | 0.00224148 | 2.25E-11 |  | 2,836 | 374,441 | -0.0389942 | 0.0310751 | 0.209538 |
| 48 | rs34284484 | G | T | -0.0123129 | 0.002169 | 1.37E-08 |  | 2,836 | 374,441 | 0.00891108 | 0.0303043 | 0.768718 |
| 49 | rs34726834 | T | C | 0.0149657 | 0.00227206 | 4.49E-11 |  | 2,836 | 374,441 | -0.0280407 | 0.02932 | 0.338888 |
| 50 | rs35408430 | T | C | -0.0213322 | 0.00206739 | 5.82E-25 |  | 2,836 | 374,441 | -0.0258168 | 0.0279103 | 0.354969 |
| 51 | rs3814995 | T | C | -0.0126428 | 0.00211677 | 2.33E-09 |  | 2,836 | 374,441 | -0.0574369 | 0.0282186 | 0.0418081 |
| 52 | rs3890624 | G | A | 0.0112736 | 0.00203846 | 3.19E-08 |  | 2,836 | 374,441 | -0.0222879 | 0.0282728 | 0.430512 |
| 53 | rs4121823 | A | T | -0.0185043 | 0.00273512 | 1.33E-11 |  | 2,836 | 374,441 | -0.0159545 | 0.0363223 | 0.660482 |
| 54 | rs4306928 | C | T | 0.0250598 | 0.00263067 | 1.63E-21 |  | 2,836 | 374,441 | -0.0232728 | 0.0361258 | 0.519435 |
| 55 | rs4364259 | A | G | 0.0165067 | 0.0024674 | 2.23E-11 |  | 2,836 | 374,441 | 0.0258365 | 0.0324364 | 0.425725 |
| 56 | rs4418728 | T | G | 0.0121203 | 0.00196937 | 7.54E-10 |  | 2,836 | 374,441 | -0.0352506 | 0.0267746 | 0.187982 |
| 57 | rs4694423 | A | C | -0.100874 | 0.00199111 | 0 |  | 2,836 | 374,441 | 0.0125676 | 0.0270771 | 0.642546 |
| 58 | rs512083 | C | T | 0.0122018 | 0.00197554 | 6.56E-10 |  | 2,836 | 374,441 | -0.0237323 | 0.026776 | 0.37544 |
| 59 | rs57459725 | G | C | -0.0176615 | 0.00289594 | 1.07E-09 |  | 2,836 | 374,441 | -0.00909138 | 0.0354953 | 0.797851 |
| 60 | rs57938057 | C | T | -0.0787942 | 0.00745585 | 4.19E-26 |  | 2,836 | 374,441 | -0.00972032 | 0.0620819 | 0.875582 |
| 61 | rs58387006 | C | A | -0.0137174 | 0.0023619 | 6.33E-09 |  | 2,836 | 374,441 | 0.013083 | 0.0317695 | 0.680479 |
| 62 | rs6003465 | C | T | -0.0119188 | 0.0020906 | 1.19E-08 |  | 2,836 | 374,441 | 0.012045 | 0.0301524 | 0.689547 |
| 63 | rs61815559 | T | A | 0.0845633 | 0.00564426 | 9.60E-51 |  | 2,836 | 374,441 | -0.0905189 | 0.148287 | 0.541577 |
| 64 | rs62012766 | C | T | -0.0181582 | 0.00269511 | 1.61E-11 |  | 2,836 | 374,441 | 0.0457468 | 0.0318007 | 0.150279 |
| 65 | rs62115743 | T | C | 0.0274464 | 0.00361625 | 3.21E-14 |  | 2,836 | 374,441 | -0.00219254 | 0.0448154 | 0.96098 |
| 66 | rs6438900 | G | C | 0.0133459 | 0.00225847 | 3.44E-09 |  | 2,836 | 374,441 | 0.0282969 | 0.0291594 | 0.331836 |
| 67 | rs6547409 | T | C | 0.0279437 | 0.00452445 | 6.57E-10 |  | 2,836 | 374,441 | 0.00668445 | 0.0502461 | 0.894166 |
| 68 | rs6671730 | A | G | -0.015115 | 0.00197958 | 2.25E-14 |  | 2,836 | 374,441 | -0.0419486 | 0.0267342 | 0.116625 |
| 69 | rs6723486 | T | C | -0.0111173 | 0.00202854 | 4.24E-08 |  | 2,836 | 374,441 | -0.00399394 | 0.0269347 | 0.88212 |
| 70 | rs6724965 | G | A | -0.0165826 | 0.00259881 | 1.76E-10 |  | 2,836 | 374,441 | 0.0407014 | 0.0326386 | 0.212386 |
| 71 | rs6834488 | T | C | -0.0146317 | 0.00199028 | 1.96E-13 |  | 2,836 | 374,441 | -0.0633908 | 0.0285524 | 0.0264083 |
| 72 | rs71601787 | A | G | 0.0419281 | 0.00210912 | 6.13E-88 |  | 2,836 | 374,441 | 0.043671 | 0.0294015 | 0.137455 |
| 73 | rs72834856 | G | T | -0.0235978 | 0.00379173 | 4.86E-10 |  | 2,836 | 374,441 | -0.0934534 | 0.0551708 | 0.0902859 |
| 74 | rs72997688 | G | A | 0.0243759 | 0.00422905 | 8.22E-09 |  | 2,836 | 374,441 | -0.011549 | 0.0520761 | 0.824492 |
| 75 | rs733454 | T | C | 0.0195549 | 0.00328005 | 2.50E-09 |  | 2,836 | 374,441 | -0.0265475 | 0.0580892 | 0.647661 |
| 76 | rs73413596 | C | T | 0.0237483 | 0.00376674 | 2.89E-10 |  | 2,836 | 374,441 | -0.0772216 | 0.0570932 | 0.176199 |
| 77 | rs7439366 | C | T | -0.0315734 | 0.00196647 | 5.20E-58 |  | 2,836 | 374,441 | -0.0130921 | 0.0270069 | 0.627841 |
| 78 | rs7522116 | T | C | -0.0126086 | 0.00199431 | 2.58E-10 |  | 2,836 | 374,441 | 0.0203033 | 0.0274364 | 0.459291 |
| 79 | rs7569755 | A | G | 0.0132378 | 0.00217782 | 1.21E-09 |  | 2,836 | 374,441 | 0.017018 | 0.0325447 | 0.601035 |
| 80 | rs7657132 | G | A | -0.0140781 | 0.00213658 | 4.43E-11 |  | 2,836 | 374,441 | 0.0152297 | 0.0289321 | 0.598613 |
| 81 | rs77924615 | A | G | -0.0144881 | 0.00251244 | 8.09E-09 |  | 2,836 | 374,441 | 0.0314102 | 0.0321849 | 0.329099 |
| 82 | rs78649910 | A | T | -0.0193309 | 0.00320205 | 1.57E-09 |  | 2,836 | 374,441 | 0.0173439 | 0.041021 | 0.672438 |
| 83 | rs79598313 | T | C | -0.0370228 | 0.00648419 | 1.13E-08 |  | 2,836 | 374,441 | -0.0603083 | 0.0736204 | 0.412684 |
| 84 | rs79687284 | C | G | -0.0295438 | 0.00537042 | 3.77E-08 |  | 2,836 | 374,441 | 0.0214414 | 0.101306 | 0.832381 |
| 85 | rs8018720 | C | G | -0.0383637 | 0.0025687 | 1.95E-50 |  | 2,836 | 374,441 | 0.0197521 | 0.037324 | 0.596661 |
| 86 | rs804281 | G | A | 0.0157516 | 0.00199025 | 2.48E-15 |  | 2,836 | 374,441 | 0.00183936 | 0.0289363 | 0.949316 |
| 87 | rs8063565 | C | G | 0.0123302 | 0.00221722 | 2.68E-08 |  | 2,836 | 374,441 | -0.0408211 | 0.0294434 | 0.165617 |
| 88 | rs8091117 | A | C | -0.02514 | 0.0039672 | 2.34E-10 |  | 2,836 | 374,441 | 0.0952021 | 0.0496731 | 0.055293 |
| 89 | rs8107974 | T | A | 0.0386164 | 0.00369585 | 1.49E-25 |  | 2,836 | 374,441 | -0.0427374 | 0.0542957 | 0.43121 |
| 90 | rs8121940 | G | C | -0.037716 | 0.00247344 | 1.69E-52 |  | 2,836 | 374,441 | -0.00194503 | 0.0316 | 0.95092 |
| 91 | rs8181687 | A | G | 0.0116958 | 0.00198761 | 4.00E-09 |  | 2,836 | 374,441 | 0.0334441 | 0.0270946 | 0.217074 |
| 92 | rs9409266 | A | G | -0.0184021 | 0.00283662 | 8.74E-11 |  | 2,836 | 374,441 | -0.0552893 | 0.035692 | 0.121366 |
| 93 | rs9467550 | G | A | -0.0188955 | 0.00319549 | 3.36E-09 |  | 2,836 | 374,441 | -0.0769186 | 0.0425893 | 0.0709104 |
| 94 | rs9476310 | T | C | 0.0117234 | 0.00197015 | 2.67E-09 |  | 2,836 | 374,441 | 0.018012 | 0.0268546 | 0.502398 |
| 95 | rs964184 | C | G | 0.0431884 | 0.00289883 | 3.37E-50 |  | 2,836 | 374,441 | -0.0140864 | 0.0380621 | 0.711315 |

SNP, single nucleotide polymorphism; EA, effect allele; OA, other allele; SE, standard error.

**Supplementary Table S5** The results of MR-Egger intercept analysis

| **Exposure** | **Outcome** | **Egger_intercept** | **SE** | ***P* value** |
| --- | --- | --- | --- | --- |
| Serum 25-Hydroxyvitamin D levels | Autoimmune thyroiditis | -0.009830044 | 0.013114595 | 0.455417703 |
| Serum 25-Hydroxyvitamin D levels | Autoimmune hyperthyroidism | -0.011195775 | 0.007053445 | 0.115844084 |
| Serum 25-Hydroxyvitamin D levels | Graves disease | 0.002259952 | 0.005975018 | 0.706119082 |

**Supplementary Table S6** The results of heterogeneity analysis

| **Exposure** | **Outcome** | **Method** | **Q** | **Q_df** | **Q_*P* val** |
| --- | --- | --- | --- | --- | --- |
| Serum 25-Hydroxyvitamin D levels | Autoimmune thyroiditis | MR Egger | 84.48986179 | 93 | 0.724018775 |
| Serum 25-Hydroxyvitamin D levels | Autoimmune thyroiditis | Inverse variance weighted | 85.05168686 | 94 | 0.734185295 |
| Serum 25-Hydroxyvitamin D levels | Autoimmune hyperthyroidism | MR Egger | 99.51977335 | 93 | 0.302946099 |
| Serum 25-Hydroxyvitamin D levels | Autoimmune hyperthyroidism | Inverse variance weighted | 102.21585 | 94 | 0.264057303 |
| Serum 25-Hydroxyvitamin D levels | Graves disease | MR Egger | 110.7877626 | 93 | 0.100716357 |
| Serum 25-Hydroxyvitamin D levels | Graves disease | Inverse variance weighted | 110.9581859 | 94 | 0.111736495 |

**Supplementary Table S7** Literature report of SNPs in serum 25-hydroxyvitamin D levels

| **SNP** | **Report** | **Source** |
| --- | --- | --- |
| rs2131925 | Angiopoietin-like 3 (rs2131925) is associated with hypertension and may play a direct role in the arterial wall. | DOI: 10.1002/mgg3.450 |
| rs1047891 | The rs1047891 is a functional variant located in the CPS1 gene. The A allele of CPS1 is a protective factor against liver fibrosis. | DOI: 10.1038/s41598-022-06614-9 |
| rs7439366 | The rs7439366 is located in the UGT2B7 gene and explains variability in free mescaline and mescaline pharmacokinetics in Chinese pediatric and adult kidney transplant recipients. | DOI: 10.1111/bcpt.13743 |
| rs4418728 | The rs4418728 (G/G) carriers are associated with an increased risk of oral and pharyngeal cancers. | DOI: 10.3390/diagnostics10110982 |
| rs964184 | The rs964184 variant of the ZNF259 gene is associated with dyslipidemia, coronary artery disease, and metabolic syndrome in a young population. | DOI: 10.3389/fnut.2022.885256 |
| rs8018720 | The rs8018720 is located in the SEC23A gene and is significantlyted with 25-hydroxyvitamin D levels. | DOI: 10.1038/s41467-017-02662-2 |
| rs1532085 | The rs1532085 in the liver lipase gene (LIPC) is associated with lipid levels. Individuals carrying the G allele have lower levels of total cholesterol, triglycerides, low-density lipoprotein (LDL) cholesterol, apolipoprotein A1, and apolipoprotein B in the Han Chinese. | PMID: 31938311 |
| rs1800588 | Hepatic lipase gene rs1800588 variant is associated with an increased risk of coronary artery disease. | DOI: 10.1080/15257770.2021.1892130 |
| rs77924615 | In the analysis across ethnicities, the strongest association with eGFR decline is rs77924615 located near UMOD/PDILT. Each time it replicated a G allele, eGFR declined 0.30% faster yearly. | DOI: 10.1681/ASN.0000000000000170 |
| rs11076175 | The rs11076175 on the CETP gene is significantly associated with low HDL-C levels. | DOI: 10.1371/journal.pone.0064191 |
